# Nitrogen-doped carbon nano-onions/polypyrrole nanocomposite based low-cost flexible sensor for room temperature ammonia detection

**DOI:** 10.1038/s41598-024-57153-4

**Published:** 2024-04-04

**Authors:** Shiv Dutta Lawaniya, Sanjay Kumar, Yeontae Yu, Kamlendra Awasthi

**Affiliations:** 1 Department of Physics, Malaviya National Institute of Technology Jaipur, Jaipur, 302017 Rajasthan India; 2 Division of Advanced Materials Engineering, Jeonbuk National University, 567, Baekje-Daero, Deokjin-Gu, Jeonju, 54896 South Korea

**Keywords:** Sensors and biosensors, Nanoscale devices

## Abstract

One of the frontier research areas in the field of gas sensing is high-performance room temperature-based novel sensing materials, and new family of low-cost and eco-friendly carbon nanomaterials with a unique structure has attracted significant attention. In this work, we propose a novel low-cost flexible room temperature ammonia gas sensor based on nitrogen-doped carbon nano-onions/polypyrrole (NCNO-PPy) composite material mounted low-cost membrane substrate was synthesized by combining hydrothermal and in-situ chemical polymerization methods. The proposed flexible sensor revealed high sensing performance when employed as the sensing material for ammonia detection at room temperature. The NCNO-PPy ammonia sensor exhibited 17.32% response for 100 ppm ammonia concentration with a low response time of 26 s. The NCNO-PPy based flexible sensor displays high selectivity, good repeatability, and long-term durability with 1 ppm as the lower detection limit. The proposed flexible sensor also demonstrated remarkable mechanical robustness under extreme bending conditions, i.e., up to 90° bending angle and 500 bending cycles. This enhanced sensing performance can be related to the potential bonding and synergistic interaction between nitrogen-doped CNOs and PPy, the formation of defects from nitrogen doping, and the presence of high reactive sites on the surface of NCNO-PPy composites. Additionally, the computational study was performed on optimized NCNO-PPy nanocomposite for both with and without NH_3_ interaction. A deeper understanding of the sensing phenomena was proposed by the computation of several electronic characteristics, such as band gap, electron affinity, and ionization potential, for the optimized composite.

## Introduction

Industry growth reflects a nation's overall strength and is correlated with economic growth. But, as the industry has grown quickly, more and more hazardous gases have been produced, endangering both human health and the environment^1–3^. Among these, ammonia (NH_3_), a colorless pollutant gas with a pungent odor, is commonly widespread in various industrial, agricultural, and commercial applications. Ammonia also plays a key role in detecting food deterioration because dried aquatic products, including meat products, fish, and shrimp will smell like ammonia after the continual breakdown of the food protein by microorganisms^4,5^. According to the Occupational Safety and Health Administration (OSHA), the permissible limit for human exposure is 25 ppm NH_3_ for 8 hours^6^. According to studies, inhaling ammonia gas over an extended period can cause serious respiratory problems, irreparable cardiac damage, and a variety of diseases^7^. So, it is essential to develop ammonia detection devices using a low-cost, practical, and effective technique. Their ability for real-time monitoring of ammonia leaks enables early intervention, lowering the risk of accidents, injuries, and adverse environmental effects^8^. In addition, the fabrication of flexible devices and sensors has also increased because of the growing significance of wearable and handheld portable sensors^9^. Their high mechanical strength and lightweight nature of flexible gas sensors allow their application in various areas, including health monitoring, food safety, environmental protection, etc.^10^.

In recent years, numerous ammonia sensors have been fabricated using a variety of gas-sensing materials, including conductive polymers, metal oxides, transition metal dichalcogenides, carbon nanomaterials, and metal hybrid materials^11^. However, they are impractical for practical use due to their high working temperatures, low selectivity, and difficult preparation. Organic conducting polymers, such as polypyrrole (PPy), polyaniline (PANI), polythiophene (PTh), and poly(3,4-ethylene dioxythiophene) (PEDOT), are the best candidates for ammonia sensors because they have low operating temperature, good sensitivity and compatibility with flexible substrates^12–14^. Among them, PPy has shown intriguing characteristics for detecting ammonia gas due to its inexpensive nature, environmental stability, good electrical conductivity, and controllable doping/dedoping processes^15,16^. However, it also exhibits some drawbacks, including low response, long response time, and poor damp-heat stability^17^. To overcome these problems, researchers have incorporated other highly efficient sensing materials into PPy matrix to enhance the gas sensing performance.

Recent investigations suggest that carbon-based nanomaterials such as carbon nanotubes (CNTs), graphene oxide (GO), reduced graphene oxide (rGO), etc., are widely exploited as filler material for gas sensing due to their high surface area, surface active sites, high chemical stability, fast charge transfer phenomenon, and good electrical conductivity^18^. Seak et al.^19^ have reported polypyrrole/ multi-walled carbon nanotubes composites based ammonia sensors, and the results show enhancement in the gas sensing performance compared to pure polypyrrole. Du et al.^20^ synthesized single-walled carbon nanotube/polypyrrole/phenylalanine core–shell nanorods via simple one-port emulsion polymerization. The results illustrate room temperature detection ability of sensor with high response and selectivity. Tiwari et al.^21^ presented a PPy/rGO thin-film-based NH_3_ sensor and showed improved sensor durability and response after incorporating rGO. Our group also reported PPy/MWCNT and PPy/f-MWCNT based room temperature ammonia sensors, which exhibit superior gas sensing performance compared to bare polypyrrole^22,23^. However, the conventional carbon nano-materials based sensor has several issues, including insensitivity at low target gas concentrations, long response/recovery time, and poor selectivity. To rectify these problems, new nano-carbons such as carbon nano-onions (CNOs), carbon dots, carbon aerogels, etc. have been synthesized, which are low-cost and possess high surface area and good electrical conductivity^24^. Among diverse carbon nanostructures, carbon nano-onions (CNOs) are increasingly being used in various applications, including gas sensing, due to their remarkable physiochemical properties^25^. CNOs are multi-layered, homocentric fullerenes having a solid or hollow internal core surrounded by concentric graphene sheets^26^. CNOs possess very high surface area and good electrical properties, making them suitable candidates for gas sensing.

In this context of designing a low-cost and highly efficient flexible gas sensor, we report a room temperature flexible ammonia sensor based on nitrogen-doped carbon nano-onions/polypyrrole (NCNO-PPy) composite synthesized via in-situ chemical oxidative polymerization method. The carbon nano-onions were synthesized by flame pyrolysis method using waste oil as precursor, and nitrogen doping was performed through hydrothermal method taking urea as nitrogen precursor. The materials were characterized by FESEM, Raman, FT-IR, BET, and XRD. The gas sensing measurements of prepared flexible sensors were examined at room temperature for various concentrations of ammonia, demonstrating enhanced sensing performance of NCNO-PPy composite compared to pure polypyrrole. The proposed flexible sensor shows a high response towards ammonia gas along with rapid response time, good selectivity, and a lower limit of detection. Additionally, the bending properties of flexible sensor prepared on low-cost membrane substrate exhibit no apparent shift in response even under bending conditions, demonstrating the sensor strong mechanical robustness. This work makes significant progress in the design of low-cost flexible room temperature sensors for real-time ammonia gas monitoring. To the best of our knowledge, no previous research has reported on the fabrication of flexible gas sensors using low cost carbon nano-onions.

## Materials and methods

### Materials

Pyrrole (98%, Sigma Aldrich), FeCl_3_ (98%, CDH) and urea (99%, CDH) are utilized in the experiments. Alpha-terpineol and PVDF are purchased from Alpha-Aesar. All raw materials used are of analytical grade quality and used without additional purification.

### Preparation of carbon nano-onions (CNOs)

Following the previously reported flame pyrolysis approach, carbon nano-onions (CNOs) were synthesized from used frying oil^27^. In a typical procedure, cotton wick was used to ignite waste frying oil in a clay lamp. For the purpose of collecting carbon soot, a glass beaker was flipped upside down and placed on top of the flame. To get unburned oil and volatile contaminants out of the collected soot, it was annealed for two hours at 500 °C in an inert atmosphere.

### Hydrothermal synthesis of nitrogen doped-carbon nano-onions (NCNOs)

The CNO (350 mg) was dispersed in 60 mL distilled water via sonication for one hour. Then, 700 mg urea was added, and the suspension was stirred for 1 h at room temperature. The obtained mixture was poured into a 80 mL Teflon-lined autoclave and kept in hot air oven at 180 °C for 12 h. After completion of reaction and cooling of autoclave at room temperature, the sample was washed with DI and dried at 60 °C.

### Synthesis of polypyrrole and N-doped carbon nano-onions composites

**Figure 1:**
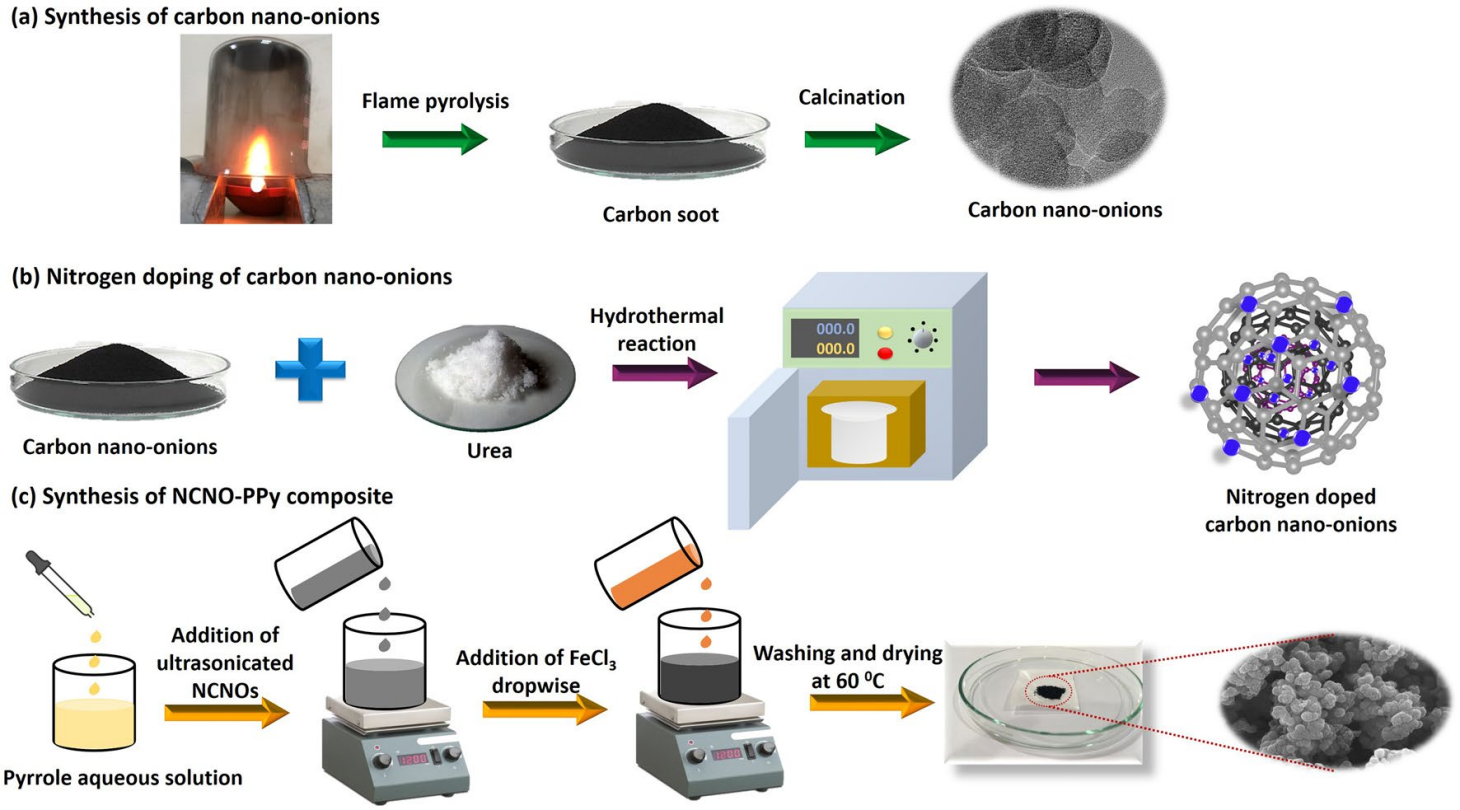
Synthesis schematic of the CNOs, NCNOs, and NCNO-PPy composite.

The NCNO-PPy nanocomposites were synthesized via in-situ chemical oxidative polymerization method taking ferric chloride (FeCl_3_) as an oxidant. Firstly, an optimized amount of CNO was dispersed into 20 mL DI with the help of sonication. The 0.1 M pyrrole was added to 50 mL of DI, and the above-prepared NCNO aqueous solution was mixed into pyrrole solution under constant stirring. The mixture was left on stirring for one hour and then, the aqueous solution of oxidant FeCl_3_ (0.05 M) was dropwise added into the Py/NCNO solution. The monomer to oxidant ratio was kept at 1:0.5. The solution was then left on stirring for 4 h for continuous polymerization. At last, the obtained black residue was washed with DI & ethanol and dried at 60 °C for overnight. For comparison, pristine PPy was also prepared by the same procedure without adding NCNO. The overall synthesis process of CNOs, nitrogen-doped CNOs and NCNO-PPy composite is shown in Fig. 1.

### Characterization

The field emission scanning electron microscopy (Nova Nano 450 FEI) was used to evaluate the surface morphology of prepared samples. The Raman spectra was observed with the help of STR 500 confocal micro raman spectrometer (AIRIX), and FT-IR spectra were captured using PerkinElmer, respectively. For the BET analysis and BJH adsorption–desorption experiments, Quanta chrome Nova Touch LX2 gas sorption analyzer was employed. X-ray photoelectron spectroscopy (XPS, An ESCA + Omicron nanotechnology spectrometer, Al K-source 1486.7 eV) was used to ascertain the chemical states of the material. The XRD spectrum of the as-prepared materials was verified by X-ray diffraction (Panalytical X’Pert Pro X-ray diffractometer) using Cu-Kα_1_ as the source (λ = 1.5406 Å).

### Fabrication of flexible gas sensor and gas sensing measurements

The inter-digitated electrodes (IDEs) were made of aluminum (Al) and e-beam deposited on a polyvinylidene fluoride (PVDF) substrate with finger spacing and thicknesses of 200 and 400 µm. An appropriate amount of powder sample and 1–2 drops of α-terpineol were combined in a mortar-pestle to create a thick slurry, which was then evenly coated on primed IDEs. Powder sample and α-terpineol were combined at a 20:1 ratio. After that, the coated IDEs were allowed to dry at ambient temperature.

In an acrylic chamber, controlled by a power supply and humidity sensor at ambient temperature (27 ± 2 °C) and relative humidity (30 ± 2%), the gas sensing measurement of the fabricated flexible sensors were investigated^28^. The 1000 ppm target gas concentration calibrated cylinder with dry air (80% N_2_ & 20% O_2_) was purchased from Ankur Gas Agency, India. Using a Keithley DMM6500 multimeter that was attached to the data-collecting system, the sensor resistance was measured. The response computed as (∆R/Ra) × 100% was used to evaluate the sensor's performance. Here, Ra and Rg are defined as the sensor's resistance in target gas and ambient air, respectively.

## Results and discussion

### Material characterizations

**Figure 2:**
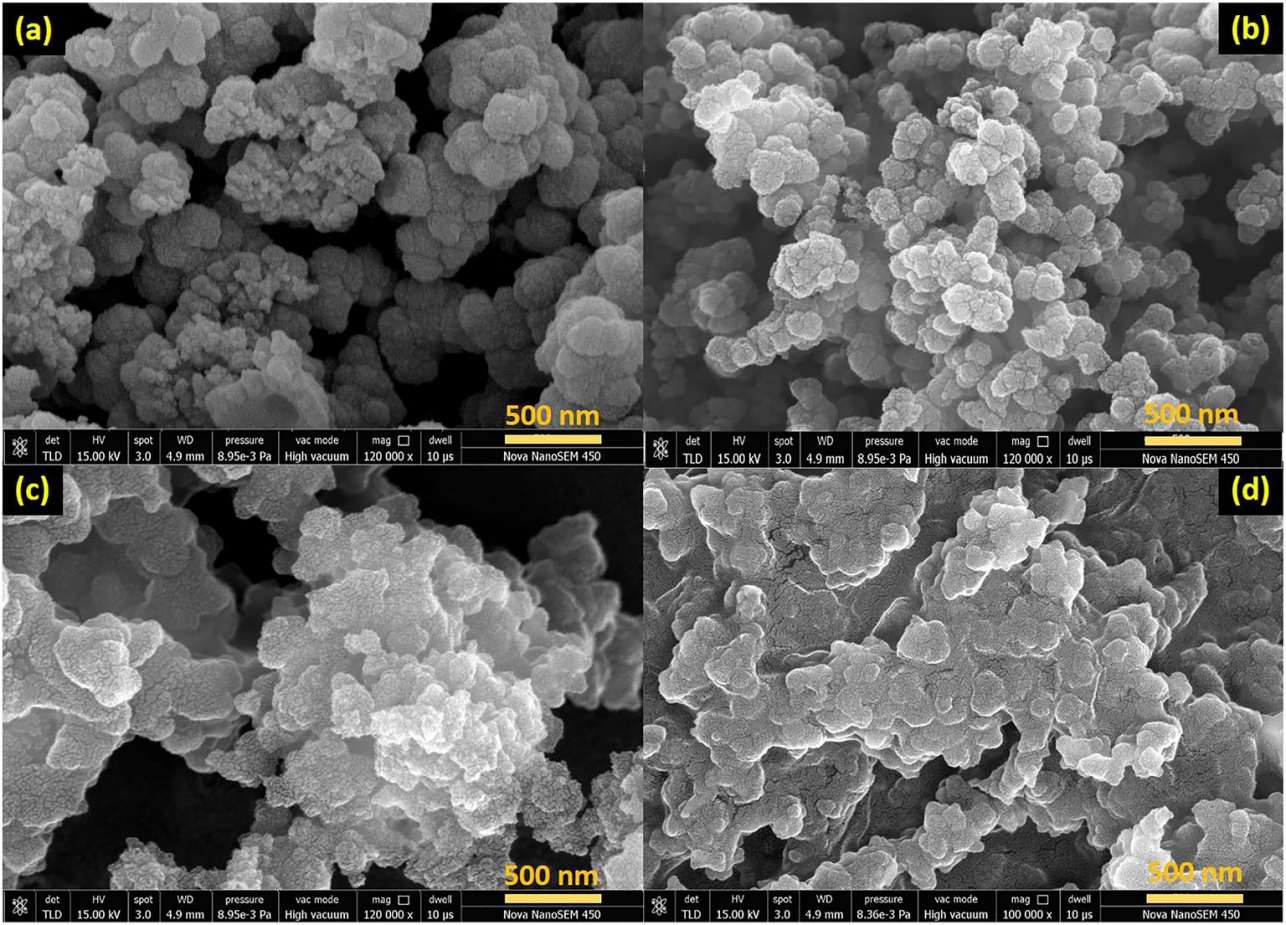
FESEM images of (**a**) PPy, (**b**) 5 wt% CNO-PPy, and (**c**-**d**) 5 wt% NCNO-PPy composite.

The morphological studies of pure PPy, 5 wt% CNO-PPy, and 5 wt% NCNO-PPy were characterized with FESEM and shown in Fig. 2. The SEM images of pure PPy exhibit random spherical structure with diameter ranging between 100 to 200 nm (Fig. 2a). The irregular size, random order, and aggregation of PPy particles demonstrate their lower activation energy and gas molecule collecting capacity, which is inferior to the gas sensing aspect. Figure 2b shows the FESEM image of 5 wt% CNO-PPy composite while FESEM images of 5 wt% NCNO-PPy are shown in Fig. 2c,d. The morphology of CNO-PPy composite exhibits coverage of PPy particles on the surface of CNOs. The CNOs act as a seeding surface for PPy particles to polymerize along them. In the case of NCNO-PPy composite SEM images, PPy embedding into the CNOs could be seen due to the presence of nitrogen atoms as a defect in the CNO structure that helps to form better bonding between CNOs and PPy. This type of arrangement provides good dispersion of CNOs into the PPy matrix, leading to a large number of inter-junction formations at the surface of the prepared material. The large junctions facilitate a high quantity of majority charge carriers to flow across the composite at the time of exposure of the target gas, which eventually enhances the gas sensing performance. Additionally, the EDS spectra of 5 wt% CNO-PPy and 5 wt% NCNO-PPy are shown in Figs. S1, S2. And the elemental percentage clearly shows the high amount of nitrogen in 5 wt% NCNO-PPy composite compared to 5 wt% CNO-PPy which is ascribed as the nitrogen doping in the CNO structure.

**Figure 3:**
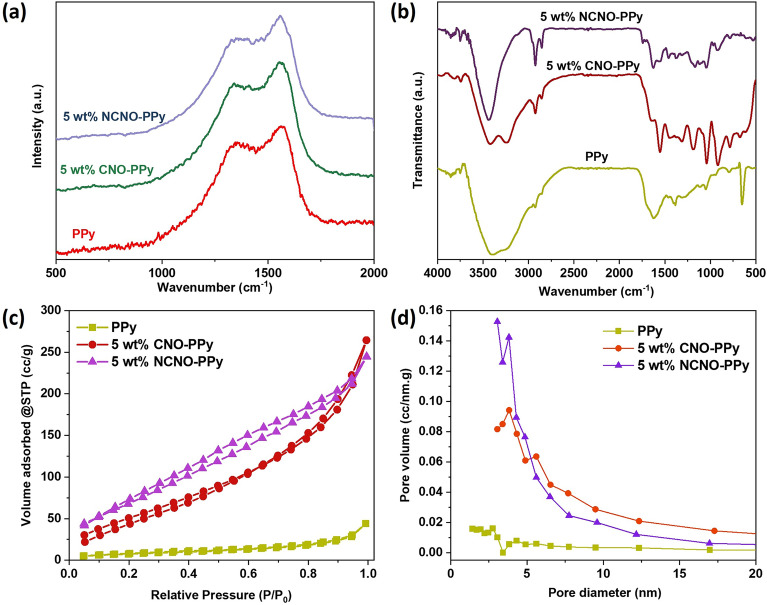
(**a**) Raman spectra, (**b**) FT-IR spectra, (**c**) N adsorption-desorption isotherms and (**d**) pore size distribution curves of PPy, 5 wt% CNO-PPy and 5 wt% NCNO-PPy composites.

Raman spectroscopy was employed to further analyze the structural characteristics of PPy and PPy-CNO composites. The Raman spectra of pure PPy, 5 wt% CNO-PPy, and 5 wt% nitrogen-doped CNO-PPy composite are shown in Fig. 3a. The intense bands in the Raman spectra of pure PPy correspond to the PPy structure's ring stretching mode, and the C=C backbone stretching vibrations were seen around 1348 cm^−1^ and 1560 cm^−1^, respectively^29,30^. The Raman spectra of CNO-PPy and NCNO-PPy illustrate two wide peaks around 1346 and 1562 cm^−1^. These peaks correspond to the merging of D and G bands with characteristic peaks of PPy^31^. There is a shifting of peaks in CNO-PPy composites compared to pure PPy, which could be due to the interaction of carbon nano-onions with the backbone of polypyrrole.

The FT-IR spectra of PPy, 5 wt% CNO-PPy, and 5 wt% NCNO-PPy are depicted in Fig. 3b. In the FT-IR spectrum of PPy, peaks at 3400, 3230, and 1619 cm^−1^ corresponds to N–H, O–H and C=C bonds stretching, respectively^32^. The peaks at 1389, 1121, and 1040 cm^-1^ are related to C=N bending, C–N stretching, and C–H bending vibrations. Additionally, the peaks at 939 and 652 cm^−1^ are attributed to C-H out of plane deformation vibration mode. The FT-IR spectra of CNOs and nitrogen-doped CNOs are shown in Fig. S3^33,34^. All the characteristic peaks of CNOs were well-matched with the literature. In the case of N doped CNO, the two most dominating peaks at 1581 and 1247 cm-1 correspond to C-N bond stretching, which confirms the addition of nitrogen into carbon nano-onions^35^. In the FT-IR spectrum of 5 wt% CNO-PPy and 5 wt% NCNO-PPy composites, all the peaks are similar to polypyrrole peaks, which indicates the good dispersion of CNOs into PPy matrix^36^. However, in the case of 5 wt% NCNO-PPy, C-N stretching peaks dominate over other peaks and O–H and N–H bond peaks are merged, which illustrates the higher incorporation and interaction of NCNO with PPy^37^.

The surface chemistry of the adsorbent, which reflects the surface area, pore volume, and pore size of the material, significantly impacts the adsorption process. Figure 3c,d displays the N_2_ adsorption/desorption isotherms and pore size distribution curves of bulk PPy, 5 wt% CNO-PPy, and 5 wt% NCNO-PPy with detailed information in Table S1. The PPy exhibits a low specific surface area of 36.2 m^2^/g and a small pore volume of 0.073 cm^3^/g due to the dense accumulation of the spherical nanoparticles and tight stacking structure, resulting in poor sensing performance^38,39^. After introducing carbon nano-onions, the surface area significantly enhanced to 225.21 m^2^/g because carbon nano-onions possess very high surface area and act as a seeding surface for PPy. When nitrogen was doped into the proposed composite, the surface area increased even more due to the nitrogen-created defects in CNOs. Additionally, the composite's hierarchical porosity and mesoporous distribution can be seen in the pore size distribution curves in Fig. 3d. Due to its large specific surface area and mesoporous structure, the as synthesized 5 wt% NCNO-PPy composite material has the proper morphology and superior surface characteristics for use as sensing material for ammonia detection.

**Figure 4:**
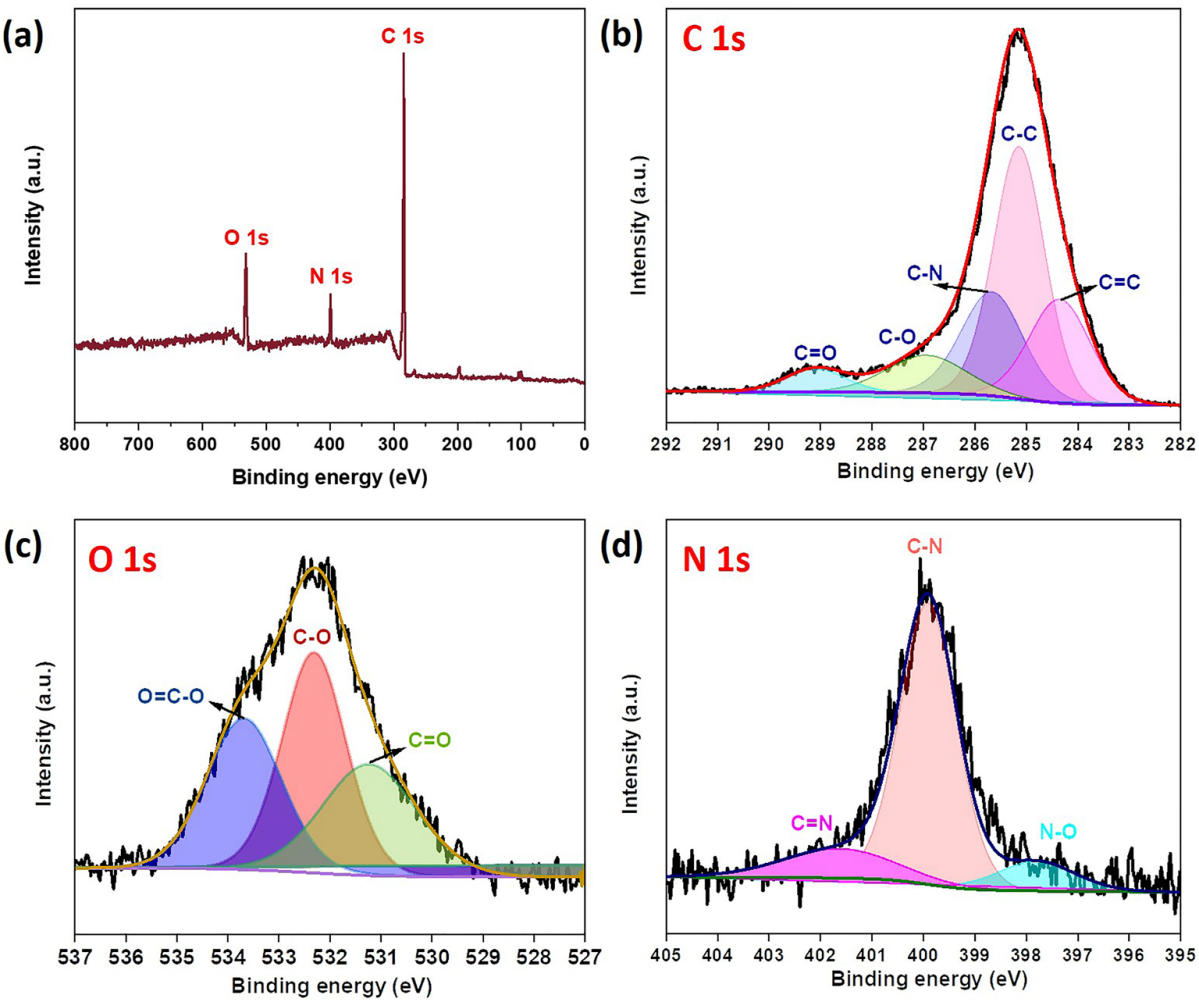
XPS analysis of 5 wt% NCNO-PPy: (**a**) survey spectra, (**b**) C 1s, (**c**) O 1s, and (**d**) N 1s high resolution spectra.

XPS spectra were used to confirm the surface elemental composition of 5 wt% NCNO-PPy. The survey spectra presented in Fig. 4a exhibit the presence of C, N and O atoms in NCNO-PPy composite. The high resolution XPS of C 1s with a range of 292–282 eV (Fig. 4b) shows five peaks by curve fitting of the C 1s spectrum. The peaks at 284.3, 285.1, 285.7, 287 and 289.1 eV correspond to the sp^2^ C, sp^3^ C, C–N/C–O, C=N/C=O and O–C=O bonds, respectively^40^. The peak corresponds to C–N confirming the in-situ polymerization of pyrrole and mixing of nitrogen-doped CNO into PPy. The O 1s spectrum could be deconvoluted into three subpeaks at 531.1, 532.3, and 533.7 eV (Fig. 4c), which might be attributed to the presence of various oxygen functionalities, including C=O, C–O, and O=C–O, respectively^41,42^. Furthermore, the pronounced N 1s peak can be split into three individual peaks at 397.8, 399.9, and 401.8 eV corresponding to N–O, C–N, and C=N, respectively, confirming the covalent bonding of pyrrole into the N doped CNO lattice (Fig. 4d)^43^. This also suggests the successful decoration of PPy on the surface of nitrogen-doped carbon nano-onions.

The XRD spectra of PPy, 5 wt% CNO-PPy, and 5 wt% NCNO-PPy are depicted in Fig. S4. In the spectrum of PPy, the broad diffraction peak appeared around 2θ = 25.04° showing the amorphous structure of polypyrrole^44^. A similar pattern was observed in the case of 5 wt% CNO-PPy and 5 wt% NCNO-PPy composite in which the peak intensity decreases for composite compared to pure PPy. The significant decrease in peak intensities illustrates the extensive coverage of carbon nano-onions by the PPy matrix, which could be seen in SEM images of CNO-PPy^45^. Additionally, the homogeneous dispersion of both components is responsible for the resemblance between the XRD patterns of CNO-PPy and pure PPy.

### Gas sensing studies

The gas sensing measurements of polypyrrole (PPy), carbon nano-onions (CNOs), and nanocomposites of PPy with CNOs and nitrogen-doped CNOs (NCNOs) were investigated for various gases, including hydrogen, carbon dioxide, carbon monoxide, ethanol, nitrogen dioxide and ammonia at room temperature. The response of a sensor is termed as the response (S) = (Rg − Ra)/Ra × 100, where Rg and Ra stand for the electrical resistance that arises from the exposure of analyte gas and air, respectively.

**Figure 5:**
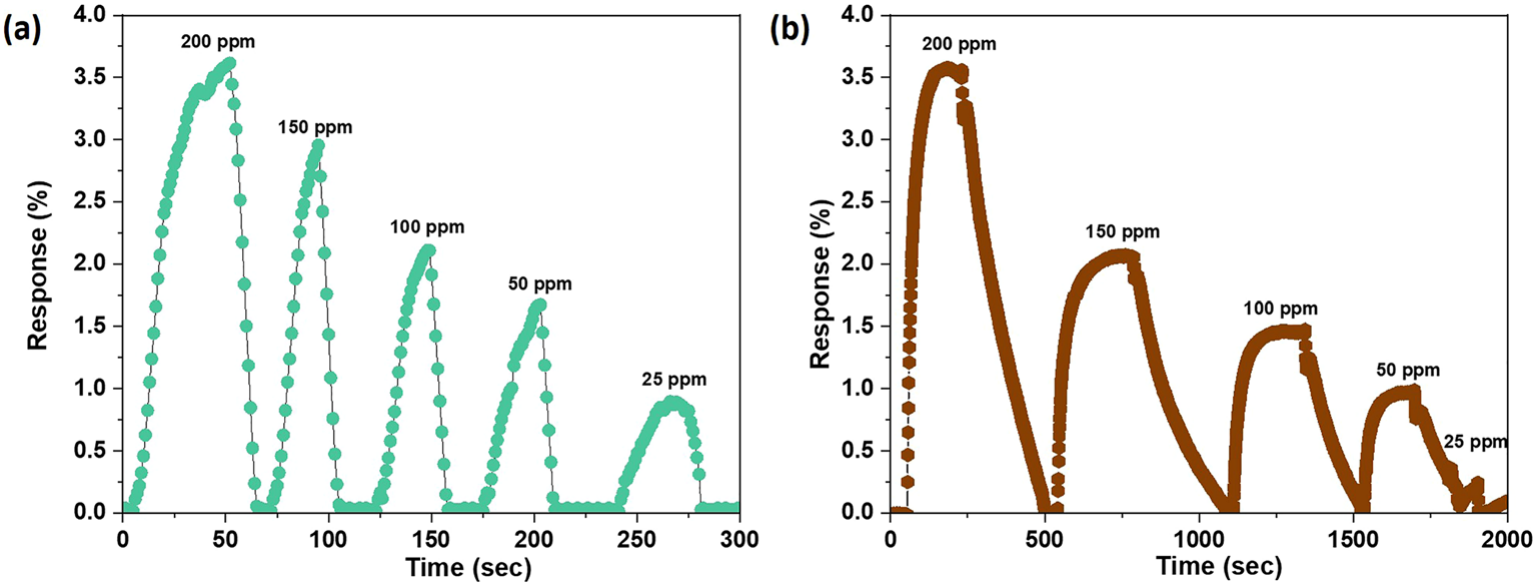
Transient response characteristics of pure (**a**) polypyrrole (PPy), (**b**) carbon nano-onions (CNOs) based flexible gas sensor.

Figure 5 shows the response curves of pure polypyrrole (PPy) and carbon nano-onions (CNOs) based flexible sensors for various concentrations of ammonia ranging between 25 and 200 ppm at room temperature. The response values of pure polypyrrole based sensor altered from 3.62 to 0.87% on going from 200 to 25 ppm NH_3_ concentration, while pure CNOs based sensor exhibited 3.57–0.23% response for the same concentration, respectively. The conductive polymer polypyrrole and carbon nano-onions exhibit p-type semiconducting behavior, and on exposure to reducing gas like ammonia, their resistance increases due to the electron–hole recombination, which takes place after electron donation of NH_3_ to sensing material.

As can be clearly seen that both sensing materials show very low response value in their pristine form because of lesser interaction of target gas molecules to sensing materials, low surface area, and poor conductivity. So, to further enhance the gas sensing performance, sensors were fabricated by utilizing PPy-CNOs nanocomposites as sensing material. To optimize the CNOs amount in PPy matrix for superior sensing performance, different combinations of PPy-CNOs composites were prepared via varying CNOs wt% between 1 and 8%.

**Figure 6:**
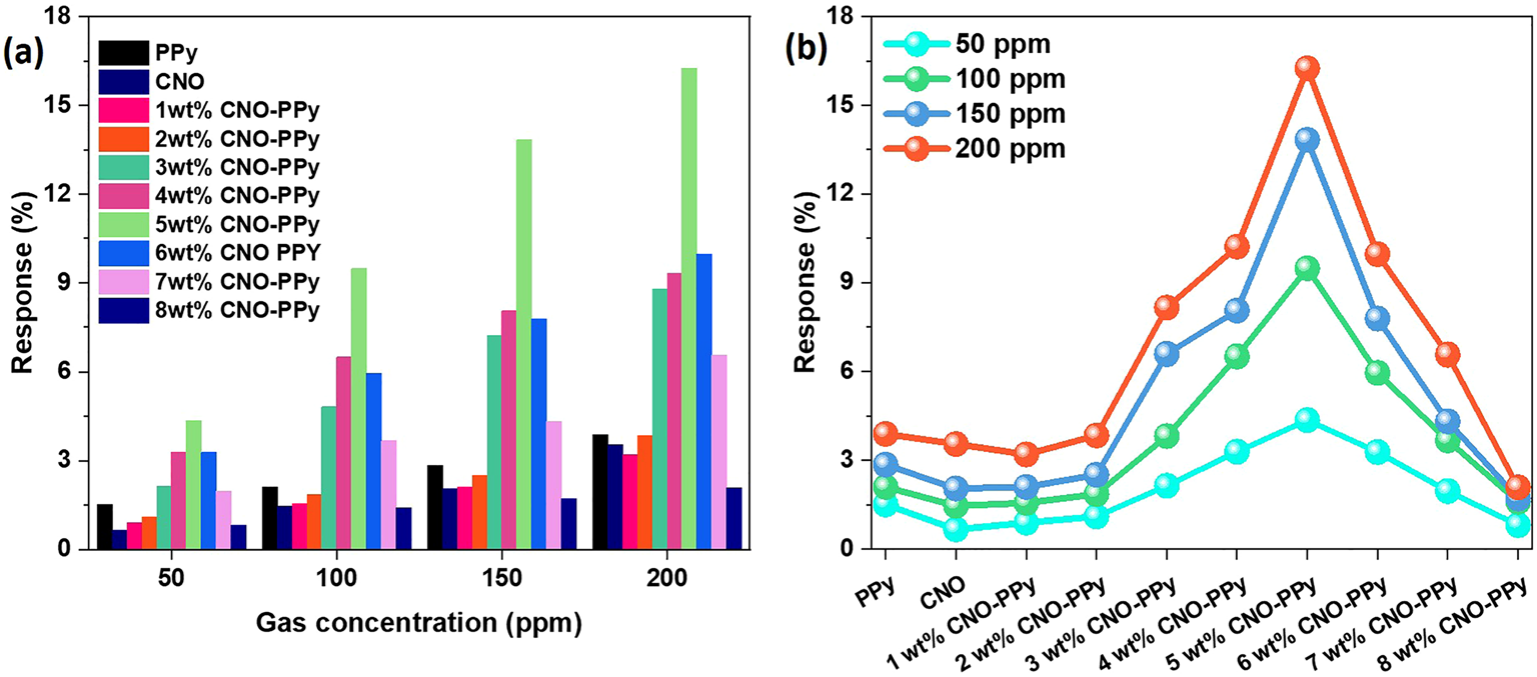
(**a**) Sensor response value comparison of pure PPy, CNO, and PPy-CNO (1–8%) nanocomposites for 50–200 ppm NH_3_ at room temperature, (**b**) response curve with the variation of CNO wt% for 50-200 ppm of NH_3_ gas at room temperature.

The various PPy-CNOs nanocomposites-based flexible sensors were tested for four concentrations (50–200 ppm) of ammonia at room temperature. Figure 6a demonstrates the response values for all prepared flexible sensors in which it can be clearly seen that 5 wt% incorporation of CNOs into PPy matrix has shown the highest response values for all concentrations of NH_3_. Initially, response values increased up to 5 wt% incorporation, and then it started decreasing on further increase the CNOs amount. The increment of response to 5 wt% could be due to the higher number of inter-junctions formation between constituents, i.e. PPy and CNOs, and a large number of target gas molecules adsorption on the surface of sensing material. The decrease in response on further adding the CNOs amount can be attributed to the larger quantity on CNOs in matrix, which can hinder the direct interaction of NH_3_ gas molecules with sensing material. A volcano curve was obtained for response values of all flexible sensors based on PPy, CNOs, and PPy-CNOs nanocomposites, as shown in Fig. 6b. After the comparative measurements, 5 wt% incorporation of CNOs into PPy was concluded as an optimized amount.

**Figure 7:**
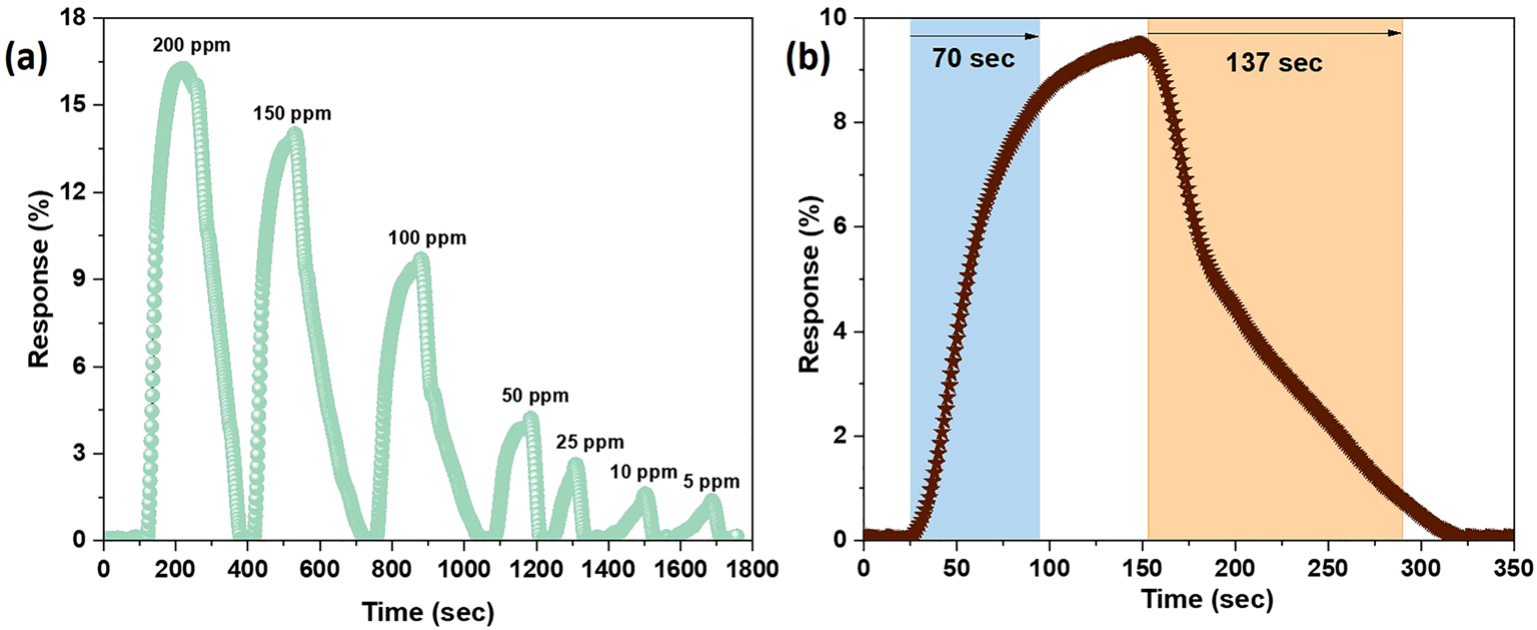
Gas sensing characteristics of 5 wt% CNO-PPy nanocomposite based flexible gas sensor (**a**) transient response curve for 5-200 ppm NH_3_, (**b**) response-recovery time curve for 100 ppm NH_3_.

The dynamic response curve of 5 wt% CNO-PPy based flexible sensor is shown in Fig. 7a, which shows the increment in response values (1.33–16.19%) on increasing the concentration of NH_3_ from 5 to 200 ppm. The enhanced gas sensing results of the 5 wt% CNO-PPy based sensor can be attributed to several factors, such as high surface area, large inter-junction formation between PPy and CNOs, and increased electrical conductivity. The response-recovery time curve for 100 ppm ammonia is shown in Fig. 7b, which shows very high response/recovery time values of 70/137 s. The higher response-recovery times could be attributed to the slow diffusion process due to the porous structure of the sensing material and temperature-dependent reaction kinetics. As can be seen that 5 wt% CNO incorporation into the PPy matrix shows superior sensing performance towards ammonia gas, but its high response-recovery times and poor detection limit constraint its application in real-time monitoring of ammonia gas.

The doping of carbon with different chemical elements, such as nitrogen, boron, phosphorus, etc. has been considered as a good strategy to increase their interaction with the target gas molecules. So, CNOs were doped with nitrogen via a hydrothermal process and further doped into PPy matrix to enhance the gas sensing performance. Since 5 wt% was concluded as the optimized quantity therefore, 5 wt% nitrogen-doped CNOs were incorporated into the PPy matrix.

**Figure 8:**
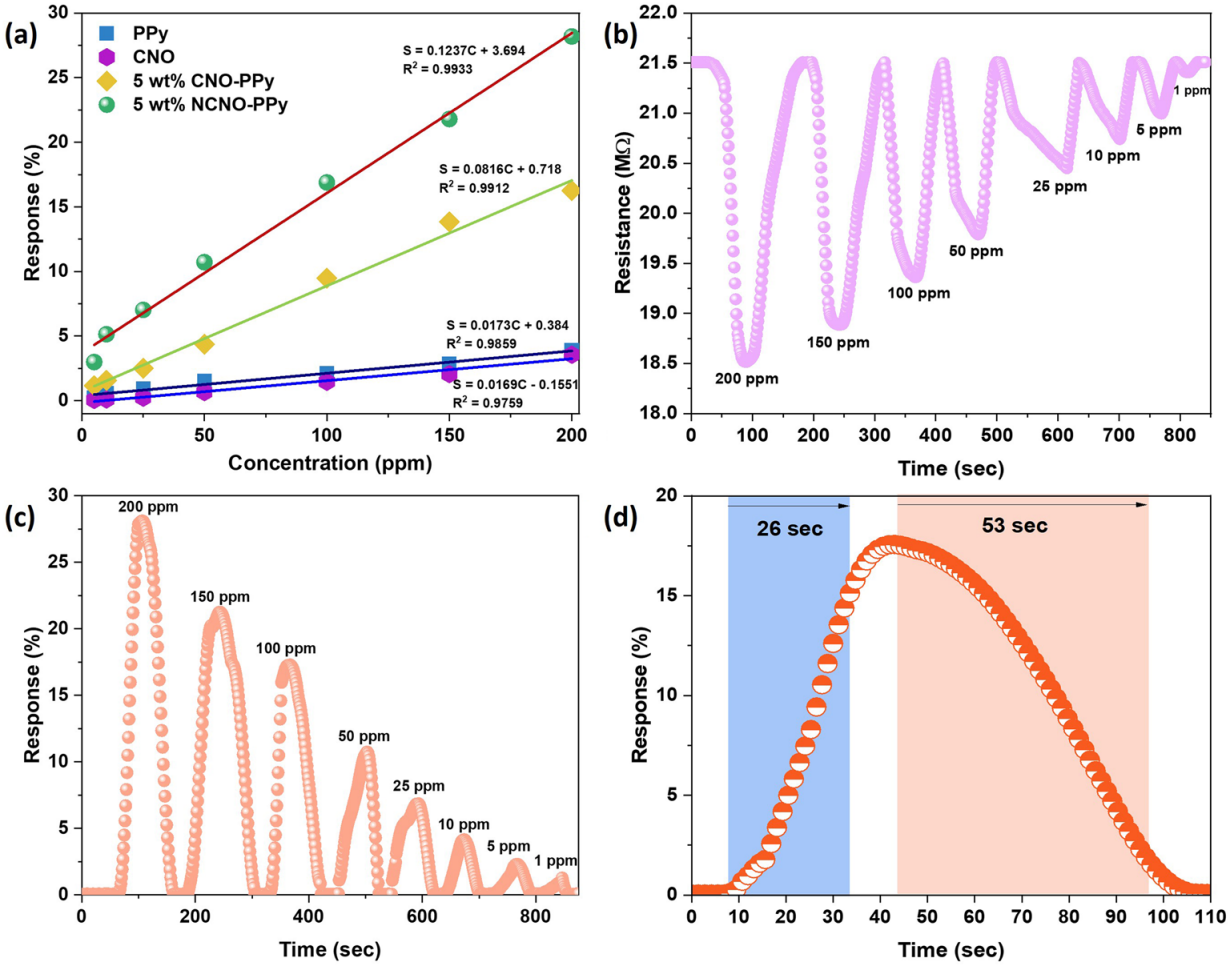
(**a**) Response-concentration fitting curve of 5 wt% NCNO-PPy compared with CNO, PPy, and 5 wt% CNO-PPy, dynamic (**b**) resistance and (**c**) response curve for 5 wt% NCNO-PPy based flexible sensor for NH_3_ at room temperature, (**d**) response-recovery time characteristics of 5 wt% NCNO-PPy for 100 ppm NH_3_.

Figure 8a demonstrates the comparison of response values for all prepared flexible gas sensors, i.e. pristine CNO, PPy, 5 wt% CNO-PPy, and 5 wt% NCNO-PPy. It also shows the calibration between concentration and response values for all sensors in which S and C correspond to (response %) and concentration, respectively. The response of the 5 wt% NCNO-PPy sensor possessed a better linear relationship toward NH_3_ concentration (R^2^ = 0.9933) than that of CNO (R^2^ = 0.9759), PPy (R^2^ = 0.9859) and 5 wt% CNO-PPy (R^2^ = 0.9912), respectively. Impressively, the sensitivity of 5 wt% NCNO-PPy sensor (0.1237 ppm^−1^) is 7.31, 7.15 and 1.51 times higher than that of CNO (0.0169 ppm^−1^), PPy (0.0173 ppm^−1^) and 5 wt% CNO-PPy (0.0816 ppm^−1^), which suggests that 5 wt% NCNO-PPy sensor has a greater ability to monitor wide detection range of NH_3_ concentration. These findings demonstrate that adding a suitable quantity of nitrogen-doped CNO can greatly improve the NH_3_ sensing capabilities of the 5 wt% NCNO-PPy sensor. Since the nitrogen-doped CNO-PPy sensor exhibits superior sensing performance than other prepared sensors, all the gas sensing measurements were investigated for 5 wt% NCNO-PPy nanocomposite-based flexible gas sensor.

The transient resistance curve of 5 wt% NCNO-PPy shown in Fig. 8b demonstrates a decrement in resistance upon exposing different concentrations of reducing ammonia gas. The proposed sensing material exhibits typical n-type semiconducting behavior on incorporating nitrogen-doped CNOs into PPy matrix. This could be due to the fact that nitrogen contains an extra electron compared to carbon, and the direct substitution of nitrogen atoms for carbon atoms in the carbon structure leads to an n-type material with localized states above the fermi level. On exposure to NH_3_ gas, it donates electrons to the sensing material, and consequently, the resistance decreases. It is obvious that for high concentrations of target gas, the change in resistance is higher, while it is also worth noticing that it can readily detect 1 ppm concentration of NH3. The dynamic response curve of 5 wt% NCNO-PPy illustrates a linear relationship between response (%) and target gas concentration. Increasing the gas concentration, higher number of gas molecules will adsorb on the surface of sensing material, which ultimately contributes to higher resistance change (Fig. 8c). The response-recovery time characteristics of the proposed sensor are calculated for 100 ppm ammonia gas concentration. As shown in Fig. 8d, the sensor exhibited 26 s as response time and 53 s as recovery time, which is very low compared to 5 wt% CNO-PPy. These results indicate that 5 wt% NCNO-PPy based sensor can be a good candidate for real-time detection of ammonia gas at room temperature.

**Figure 9:**
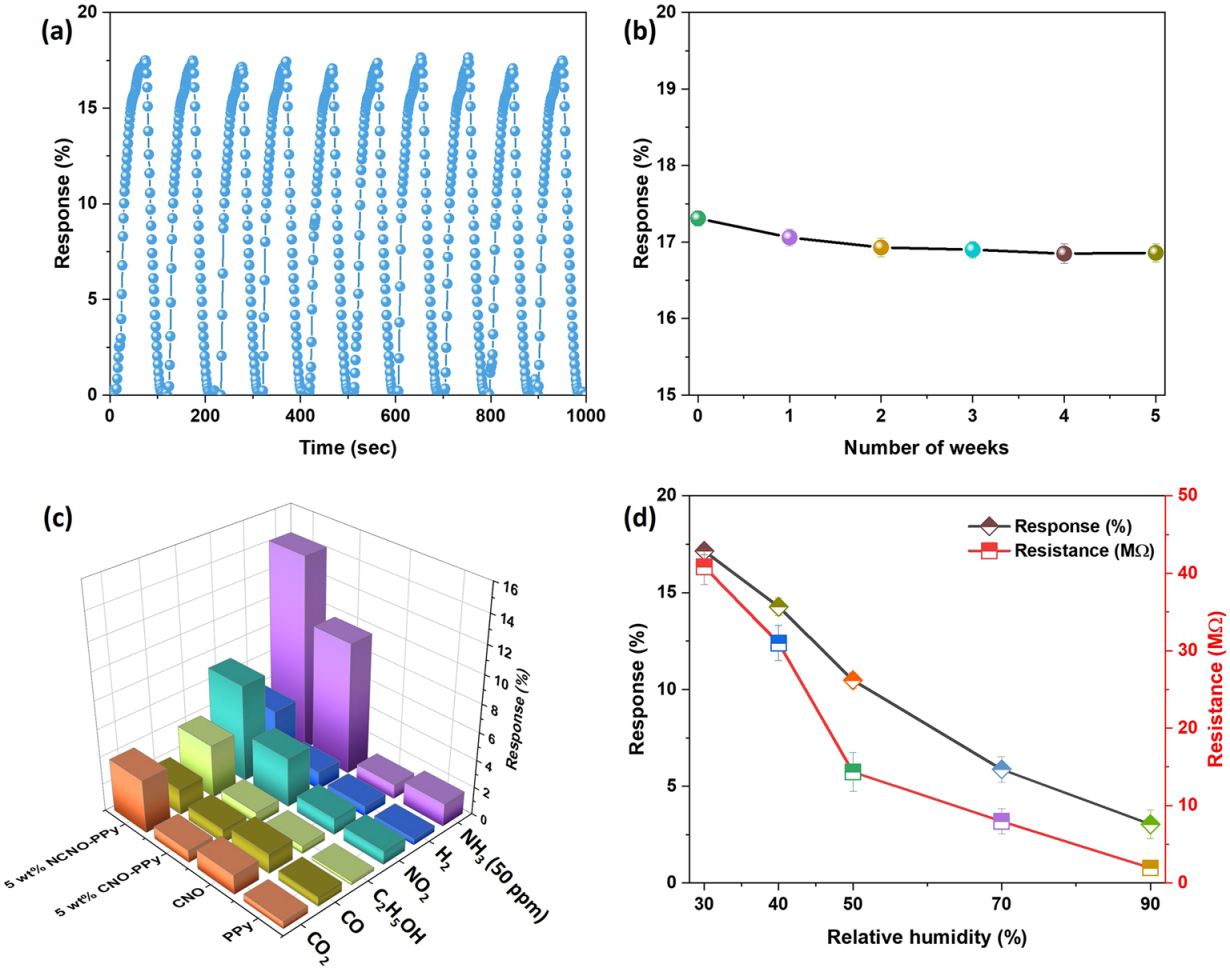
(**a**) Reproducibility of 5 wt% NCNO-PPy to 100 ppm NH_3_ up to 10 continuous cycles, (**b**) long-term stability test, (**c**) selectivity characteristics of PPy, CNO, 5 wt% CNO-PPy and 5 wt% NCNO-PPy and (**d**) response of 5 wt% NCNO-PPy to 100 ppm NH_3_ at different RH.

Repeatability characteristics are crucial in gas sensors as they demonstrate the sensor's ability to consistently produce accurate signals when used continuously. Therefore, the proposed sensor was tested for 10 successive cycles of 100 ppm ammonia gas at room temperature (Fig. 9a). The results demonstrated no considerable change in response for 10 continuous cycles with 0.04 as the standard deviation. The repeatability test was also performed on 25 ppm NH_3_ for 10 cycles, and the results show no considerable change in response values with a standard deviation of 0.11 (Fig. S5). Figure 9b demonstrated the 5 wt% NCNO-PPy sensor long-term stability over a period of five weeks. The sensory response clearly reduced up to two weeks and later reached a stable state in the following weeks. The response value retained 97% of its initial value after 5 weeks, indicating that this sensor had a respectable level of stability. The loss of unstable absorption sites and sensor aging can be attributed for the gradual decline in gas-sensing capability.

The selectivity performance of the sensor was further explored for PPy, CNO, 5 wt% CNO-PPy, and 5 wt% NCNO-PPy based flexible sensor because it works as a significant indicator in sensor practical application. As shown in Fig. 9c, the response of the above-mentioned sensor was tested for 100 ppm of various gases including carbon dioxide, carbon monoxide, ethanol, nitrogen dioxide, hydrogen, and 50 ppm ammonia. It can be clearly seen that the proposed 5 wt% NCNO-PPy nanocomposite-based sensor exhibited superior sensing performance compared to all other sensors and exhibited a higher response value than other gaseous analytes. Comparing the proposed material to other recently published sensing materials, the selectivity factor (K) was determined and listed in Table S2. In the combination of gases, a greater value of K indicated a better competence to a particular gas. Compared to these sensing materials, the 5 wt% NCNO-PPy sensor demonstrated good selectivity. Humidity significantly affects a sensor's ability to detect gases. Therefore, the effect of humidity was also investigated for 5 wt% NCNO-PPy based sensor. Figure 9d shows a decrement in response values on increasing the relative humidity values, possibly due to the diffusion and hindrance of water molecules in the interaction between sensing material and target gas.

**Figure 10:**
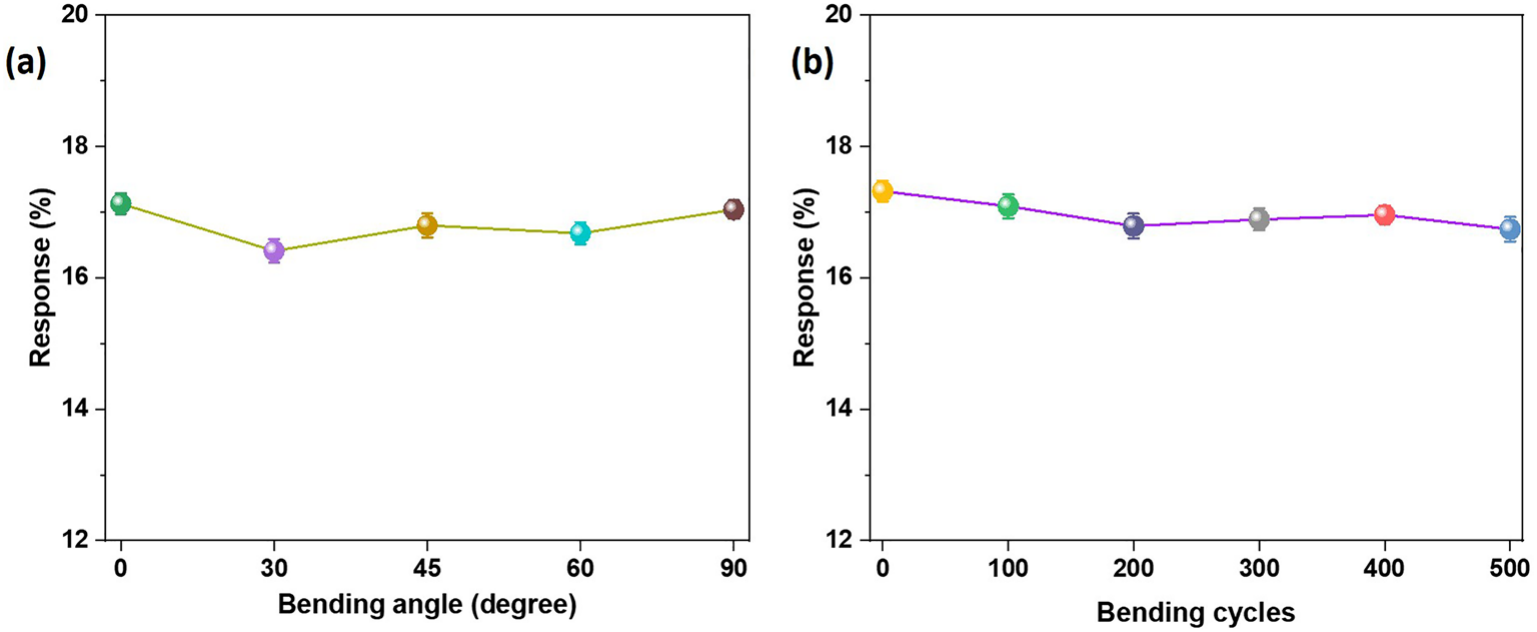
Response of 5 wt% NCNO-PPy sensor to 100 ppm NH_3_ (**a**) at different bending angles, and (**b**) at various bending cycles.

Excellent bending characteristics are projected to be one of the most essential characteristics for implementing flexible gas sensors in wearable electronics. To examine the impact of bending angles on the proposed flexible sensor, gas sensing experiments were conducted at various bending angles. The response of the flexible sensor is shown in Fig. 10a for different bending angles, which shows no significant change in response on going up to 90° angle. Additionally, the gas sensing performance of the proposed flexible sensor was evaluated for 500 cycles of bending. Figure 10b shows very less fluctuations in response values on going up to 500 bending cycles. Also, no cracks were observed while performing the bending measurements, which could be due to the fact that carbon nano-onions provide good mechanical properties to the nanocomposite. In conclusion, the bending properties of flexible gas sensors mounted on membrane-based substrates demonstrate their great degree of flexibility and robustness. In addition, the response curve of proposed flexible sensor under different bending conditions is shown in Fig. S6 (Table 1).

**Table 1:** Comparison table of gas sensing performance of carbon nano-materials/polypyrrole based room temperature ammonia sensor.

Sensing material	Substrate	Concentration (ppm)	Response	Response time (sec)	References
PPy/SWCNT	Silicon	150	2.2^a^	22	^46^
PPy-COOH-MAF-6	Alumina	100	3.27^a^	312	^47^
PPy/MWCNT/SLS	Glass	150	30^b^	228	^48^
SWCNT-PPy-PA	Glass	1	2^a^	600–700	^49^
PPy-rGO	Glass	125	13.3^b^	233	^21^
Ppy-PA/Ag-SWCNT	PDMS	3	8^a^	25	^50^
5 wt% CNO-PPy	Membrane	100	9.7^b^	70	This work
5 wt% NCNO-PPy	Membrane	100	17.32^b^	26	This work

^a^Response = Rg/Ra.

^b^Response = (Rg − Ra)/Ra × 100%.

### Ammonia sensing mechanism

Conjugated polymers are prominent semiconductor materials, yet polypyrrole (PPy) stands out for having high HOMO (highest occupied molecular orbital) and LUMO (least unoccupied molecular orbital) levels, which can be easily doped p-type doped and exhibit high degree of stability^51,52^. The gas sensing mechanism of pure polypyrrole (PPy) is hypothesized as the protonation/deprotonation process through the adsorption–desorption process of NH_3_ on the surface of PPy. On the exposure of recurring gas like NH_3_, it donates electrons and forms ammonium ion, while in the case of PPy, its resistance decreases due to the electron–hole recombination process.

In the context of the CNO-PPy ammonia sensing mechanism, the carbon nano-onions have a high number of defects and empty sites, which is beneficial for ammonia adsorption. In the synthesis process, CNOs provide a surface for pyrrole to polymerize throughout the active surface of CNO. As a result, in-situ polymerization process allows intense interaction between the CNO and PPy. The experimental results indicate that bare CNOs are capable of interacting with NH_3_ molecules, but a significantly stronger interaction occurs when CNOs are added to this PPy matrix^53^. In another way, the interaction between both nanomaterials with the target gas results in a synergistic sensing effect that improves the performance of the sensor^54^. Additionally, both nanomaterials exhibit an impressive interaction via persistent hydrogen bonding and π-π stacking^55^. This type of interaction between two constituent materials provides a high adsorption rate and reactive sites to the composite. The enhanced adsorption rate helps in the interaction of target gas molecules with sensing material surface strongly and results in a high change in resistance. Additionally, the high surface area and porous structure of CNOs, as well as its high electron mobility with PPy, help in increasing the charge transfer mechanism. As a result, it leads to an effective transduction and diffusion process for the ammonia gas molecules on the surface of composite material^56^. Ultimately, all the factors attributed to the enhancement in the gas sensing performance of CNO-PPy composite compared to pure PPy.

**Figure 11:**
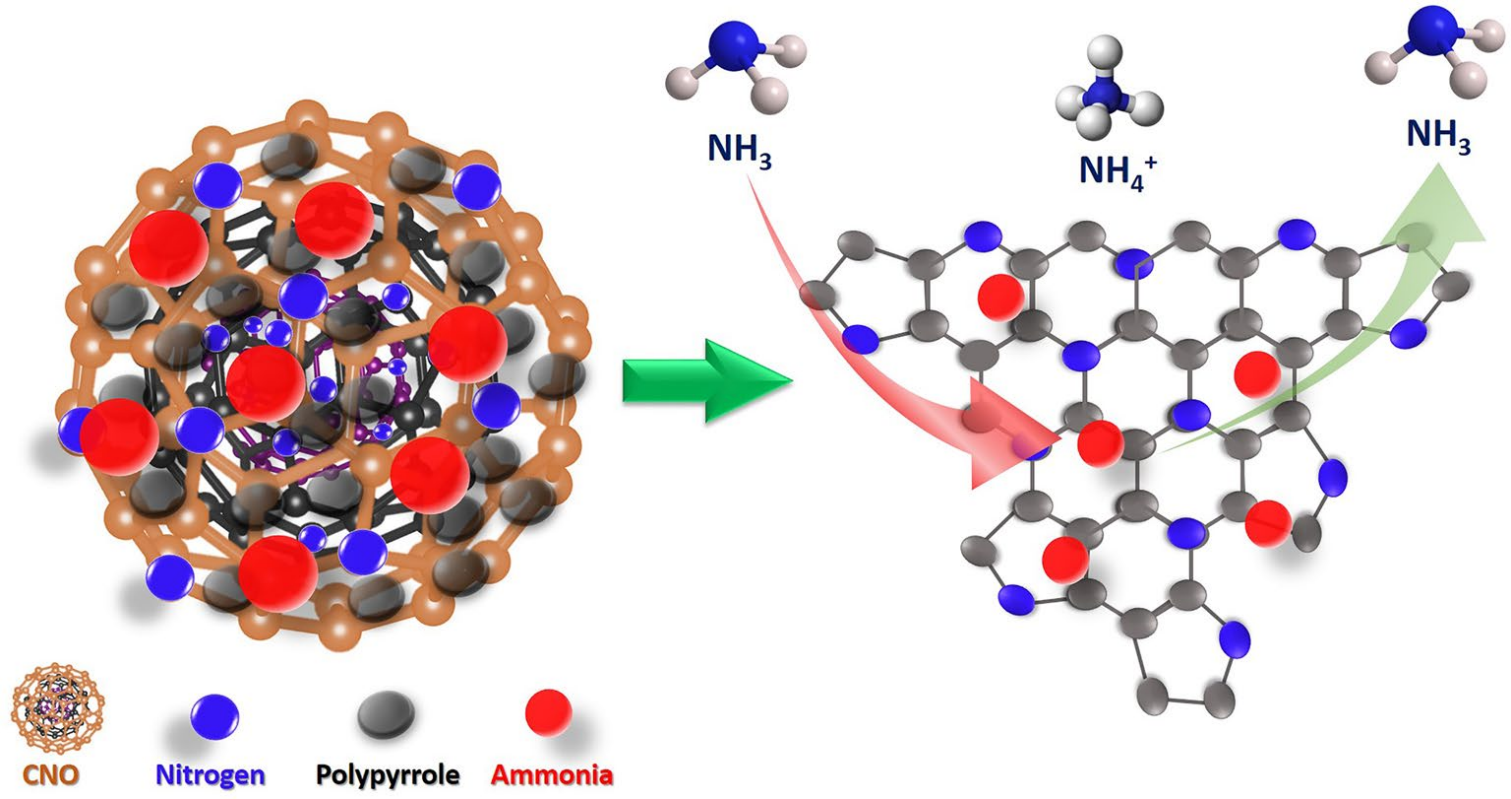
Illustration of sensing mechanism of NCNO-PPy composite based room temperature ammonia sensor.

Figure 11 illustrates the ammonia sensing mechanism of nitrogen-doped CNOs-PPy nanocomposite. The nitrogen doping in CNOs creates defects in the carbon structure and increases the surface area/reactive sites, which facilitates a large number of ammonia gas molecules on the surface. The nitrogen doping also helps in increasing the bonding between both CNOs and PPy. This severe interaction between both materials provides a continuous electrical pathway for charge carriers to move throughout the structure. The NCNO-PPy composite also shows a similar sensing mechanism to PPy in which the ammonia gas converts into ammonium ions on interaction with the sensing material. Since NH_3_ is a donor of electronic charge and the sensitive film is a receptor, because HOMO level of the gas molecule interacts with the LUMO level of the sensor. This kind of electron-donating gas is coupled to the sensitive NCNO-PPy material by π-bonds. Thus, the concentration of the majority charge carriers (holes) in the nitrogen-doped carbon nano-onions loaded with PPy changes when exposed to ammonia, which results in a considerable variation in overall sensor resistance^57^. In addition, the decrement in baseline resistance of NCNO-PPy composite compared to CNO-PPy and pure PPy can be observed in Fig. S7, which implies the higher conductivity of the proposed sensor. These results are consistent with the previously reported PPy-carbon composite based sensor, indicating that the NCNO-PPy nanocomposite has superior sensing properties than CNO-PPy and pure PPy.

### Computational study

**Figure 12:**
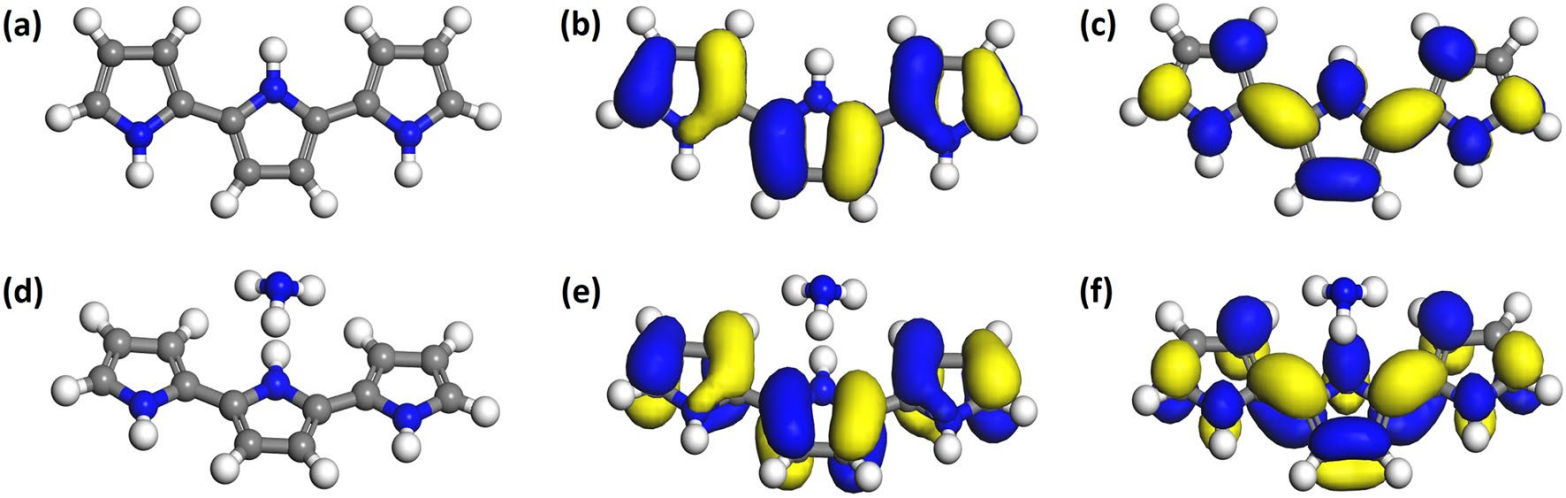
Geometrically optimized strcuture, highest occupied molecular orbitals (HOMO) diagram and lowest unoccupied molecular orbitals (LUMO) diagram for (**a**-**c**) 3Py and (**d**-**f**) ammonia adsorbed 3Py.

The DFT analysis of the PPy, PPy/CNO and PPy/NCNO composites was completed in order to better understand the sensing phenomenon employing the Materials Studio program. The detailed information about the computational methodology is presented in supplementary information. The reference optimized structure of polypyrrole is shown in Fig. S8. For the adsorption of ammonia, the particular positions were also taken into account. The geometrical optimized structure of polypyrrole and NH_3_ adsorbed PPy is shown in Fig. 12 along with their frontier molecular orbital diagrams (i.e. Highest occupied molecular orbitals (HOMO) and Lowest unoccupied molecular orbitals (LUMO), which is consistent with previous reports^58–60^. The sensing ability also highly influenced by how an oligomer's molecular orbitals interact with the analyte. So the change in bond lengths and bond angles on before and after adsorption of NH_3_ is shown in Table S3.

Further, to prove the sensing mechanism of PPy and NH_3_, the HOMO, LUMO and band gap position was examined in both cases and presented in Table S4. When ammonia is added to 3Py oligomers, their band gap value decreases. The conductance of 3Py rises upon mixing with ammonia, as seen by the band gap data of Py, which is in line with recent experimental findings. According to experimental findings, conducting polymer doping (oxidation) reduces band gap value and improves conductance. Conversely, dedoping, or decrease, has the opposite effect. We have mimicked ammonia's doping ability in the undoped form of PPy, but it can also cause reduction (dedoping) in doped conducting polymers. This reduction in resistance in 3Py is caused by an increase in electron density (accepting electron from ammonia) in the LUMO, which causes the band gap to decrease.

Further, the optimized structure of carbon nano-onions, corresponding HOMO, LUMO and nitrogen doped carbon nano-onions is presented in Figs. S12, S13. The position of HOMO, LUMO and band gap values are compiled in Table S5. For the nitrogen doping, various positions were considered along with maximum three nitrogen doping per CNO structure^61,62^. For providing information on the materials and their interaction about the target analyte, factors including HOMO, LUMO and band gap energy, electron affinity, ionization potential, electronegativity and most importantly interaction energy proves to be helpful. Material electron affinities provide information on their reactivity. Comparably, the smallest amount of energy needed to ionize the materials is known as the ionization potential. Easy ionization of the materials due to their lower ionization potential provides valuable insights on the adsorption of analytes^63–65^. Figures S14, S15 shows the NH_3_ molecule before and after interaction with the PPy-CNO and PPy-3NCNO composite, with HOMO and LUMO orbitals. Table 2 displays the computed electronic characteristics including interaction energy between the composite and ammonia molecules calculated via Eq. S1. It can be clearly seen that comparison to pure PPy, PPy-CNO composite shows higher interaction energy. Hydrogen bonding between ammonia and Py oligomers occurs because of the electronegativity differences between N in NH_3_ (partial negative end) and H in nPy (partial positive end). Further, the hydrogen bond and π-π stacking interaction between the polypyrrole and carbon nano-onions provides a continuous pathway for charge carriers and consequently increases the conductive nature. The interaction energy further increases after doping of nitrogen atom into CNOs structure. This increased energy values shows the good interaction between the composite material and target gas analyte proposing material highly sensitive nature.

**Table 2:** HOMO, LUMO, band gap, electron affinity, ionization potential, electronegativity and adsorption energies of various composites with and without ammonia adsorption.

Composite material	HOMO (eV)	LUMO (eV)	Band gap (eV)	Electron affinity (eV)	Ionization Potential (eV)	Electronegativity (eV)	ΔE (adsorption) in kcal/mol
3Py-CNO	− 3.515	− 1.821	1.694	1.821	3.515	2.668	–
3Py-CNO-NH_3_	− 3.462	− 1.824	1.638	1.824	3.462	2.643	− 29.4
3Py-1NCNO	− 5.392	− 5.156	0.236	5.156	5.392	5.274	–
3Py-1NCNO-NH_3_	− 5.351	− 5.196	0.155	5.196	5.351	5.2735	− 109.408
3Py-2NCNO	− 5.41	− 5.153	0.257	5.153	5.41	5.2815	–
3Py-2NCNO-NH_3_	− 5.325	− 5.188	0.137	5.188	5.325	5.2565	− 111.931
3Py-3NCNO	− 5.322	− 4.995	0.327	4.995	5.322	5.1585	–
3Py-3NCNO-NH_3_	− 5.219	− 5.055	0.164	5.055	5.219	5.137	− 101.745

## Conclusion

In conclusion, a flexible room-temperature ammonia gas sensor was developed employing nitrogen-doped carbon nano-onions/polypyrrole (NCNO-PPy) composite synthesized via a combination of hydrothermal method and in-situ chemical polymerization. The morphological, structural, and surface characteristics of the sensing material were examined, and fabricated flexible sensor was assessed for the detection of wide range of ammonia (1–200 ppm) at room temperature. The proposed NCNO-PPy composite based ammonia sensor showed superior sensing performance compared to bare PPy, CNOs, and CNO-PPy composite, which could be due to the high surface area (237.228 m^2^/g) and porous nature (3.04 nm pore size). The proposed NCNO-PPy based flexible ammonia sensor exhibited high gas response (17.32% response for 100 ppm NH_3_) with rapid response time (26 s) and a lower limit of detection (1 ppm). In addition, the NCNO-PPy composite sensor demonstrated great repeatability, long-term stability (5 weeks), high selectivity to ammonia among diverse interfering gases, and good sensing performance under bending conditions. Moreover, the DFT analysis yielded valuable insights into the high efficiency and sensitivity of NCNO-PPy nanocomposite for NH_3_ detection. The high-performance flexible room-temperature NH_3_ sensor developed in this work has a lot of potential in different fields, including food quality assessment, public safety, and health monitoring.

### Supplementary Information

Supplementary Information.

## Data Availability

The original contributions presented in the study are included in the article/Supplementary material; further inquiries can be directed to the corresponding author.
