# Supplementary material for: Nitrogen-doped carbon nano-onions/polypyrrole nanocomposite based low-cost flexible sensor for room temperature ammonia detection
Source: Sci Rep. 2024 Apr 4;14:7904. doi: 10.1038/s41598-024-57153-4 (PMC10991286; doi:10.1038/s41598-024-57153-4)
Supplement: Supplementary file 1 — Supplementary Information. [file 41598_2024_57153_MOESM1_ESM.docx]

**Supplementary Materials**

**Nitrogen-doped Carbon Nano-onions/Polypyrrole nanocomposite based low-cost flexible sensor for room temperature ammonia detection**

**Shiv Dutta Lawaniya^1^, Sanjay Kumar^1^,** **Yeontae Yu^2^, Kamlendra Awasthi^1*^**

*^1^Department of Physics, Malaviya National Institute of Technology Jaipur, Jaipur-302017, Rajasthan, India*

*^2^Division of Advanced Materials Engineering, Jeonbuk National University, 567, Baekje-daero, Deokjin-gu, Jeonju 54896, South Korea*

[*kawasthi.phy@mnit.ac.in](mailto:*kawasthi.phy@mnit.ac.in)


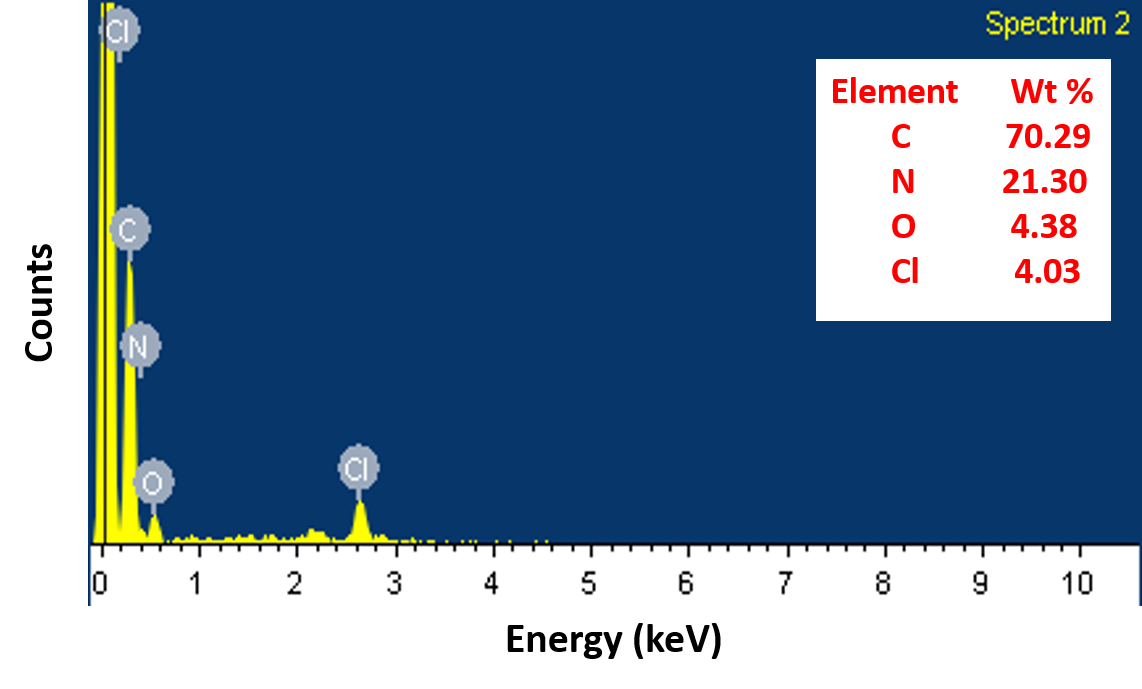


Figure S1 EDS spectra of 5 wt% CNO-PPy composite.


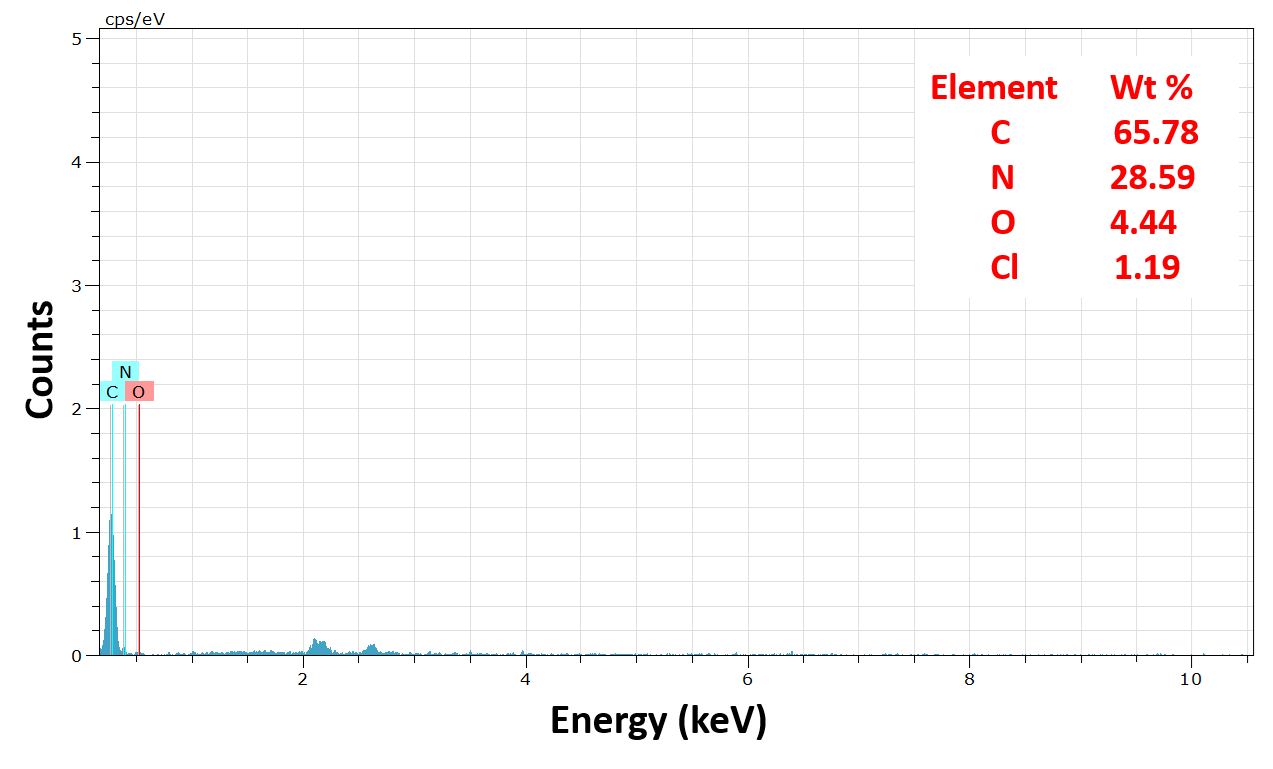


Figure S2 EDS spectra of 5 wt% nitrogen-doped CNO-PPy composite.


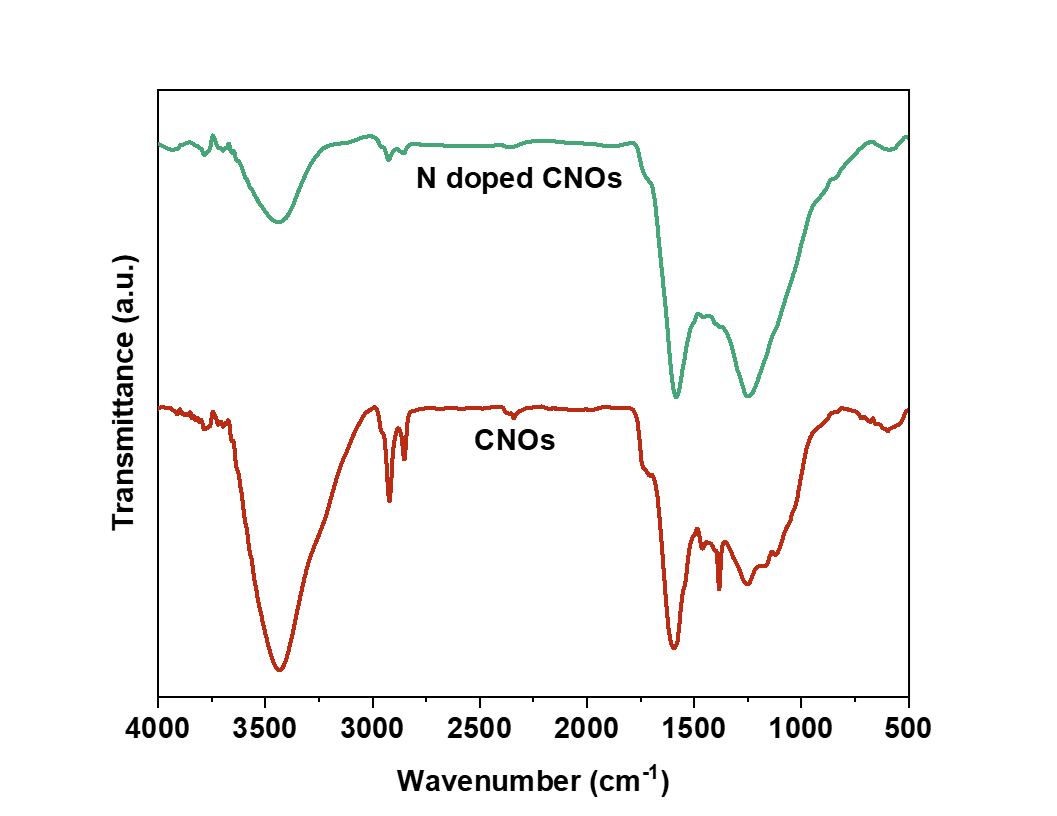


Figure S3 FT-IR spectra of CNO and nitrogen-doped CNO.

Table S1. The surface area, pore diameter, and pore volume of PPy, 5 wt% CNO-PPy and 5 wt% NCNO-PPy.

| Material | Surface area (m^2^/g) | Pore diameter (nm) | Pore volume (cm^3^/g) |
| --- | --- | --- | --- |
| PPy | 38.137 | 2.74 | 0.073 |
| 5 wt% CNO-PPy | 225.214 | 3.82 | 0.40 |
| 5 wt% NCNO-PPy | 237.228 | 3.04 | 0.31 |

Table S2. Selectivity factor (K) values of 5 wt% NCNO-PPy composite for 100 ppm concentration of target gaseous analytes with reference to ammonia.

| Interfering gas | CO_2_ | CO | C_2_H_5_OH | NO_2_ | H_2_ |
| --- | --- | --- | --- | --- | --- |
| K factor | 4.49 | 9.16 | 4.47 | 2.43 | 4.25 |


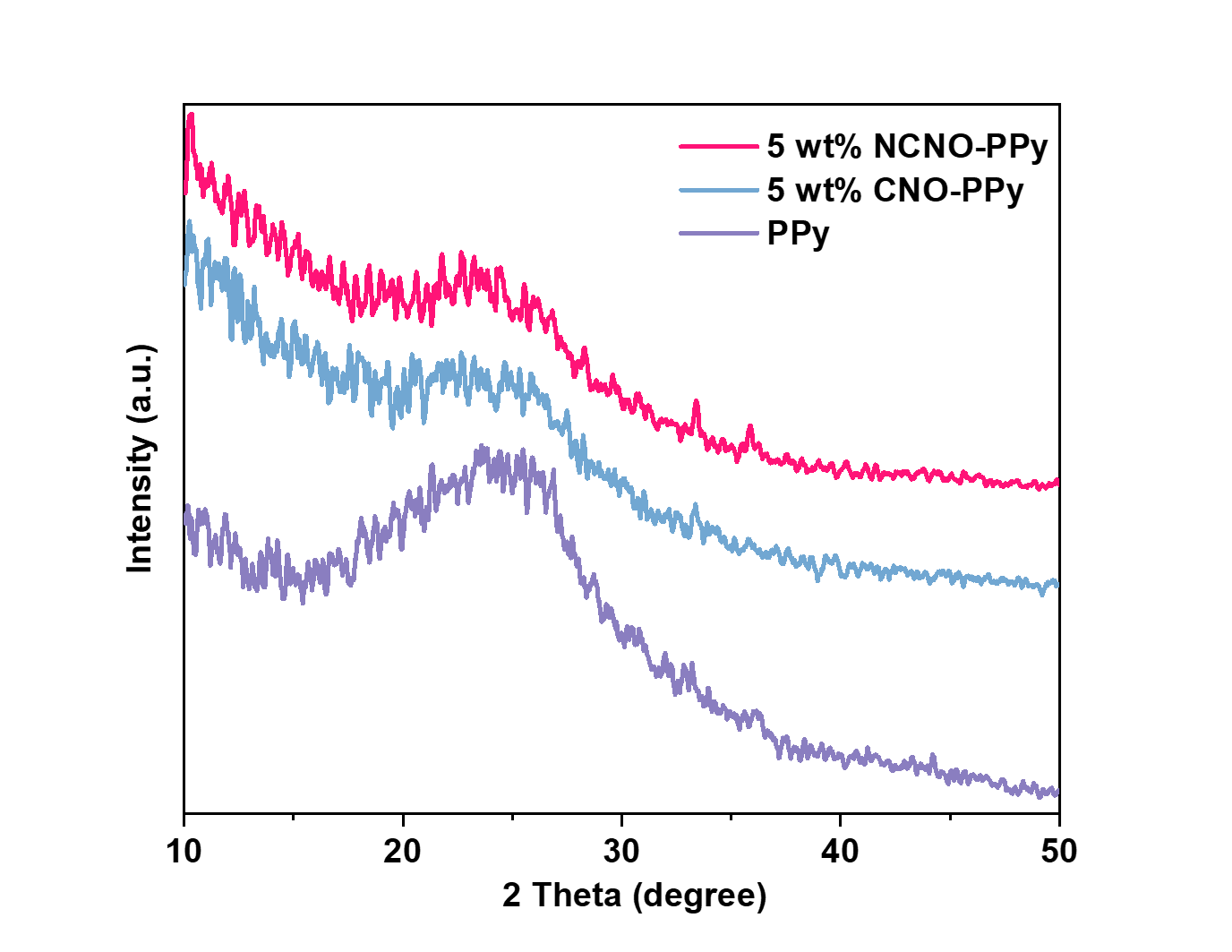


Figure S4 XRD spectra of PPy, 5 wt% CNO-PPy and 5 wt% NCNO-PPy.


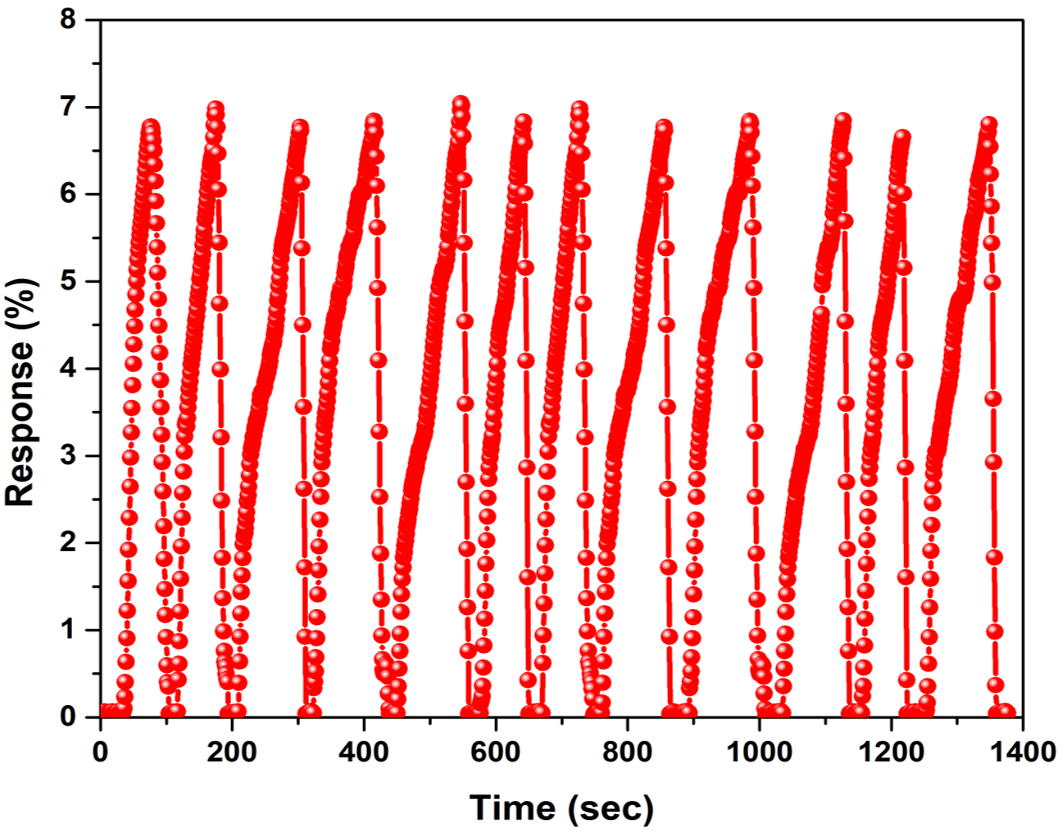


Figure S5 Reproducibility of 5 wt% NCNO-PPy to 25 ppm NH_3_ up to 12 continuous cycles.


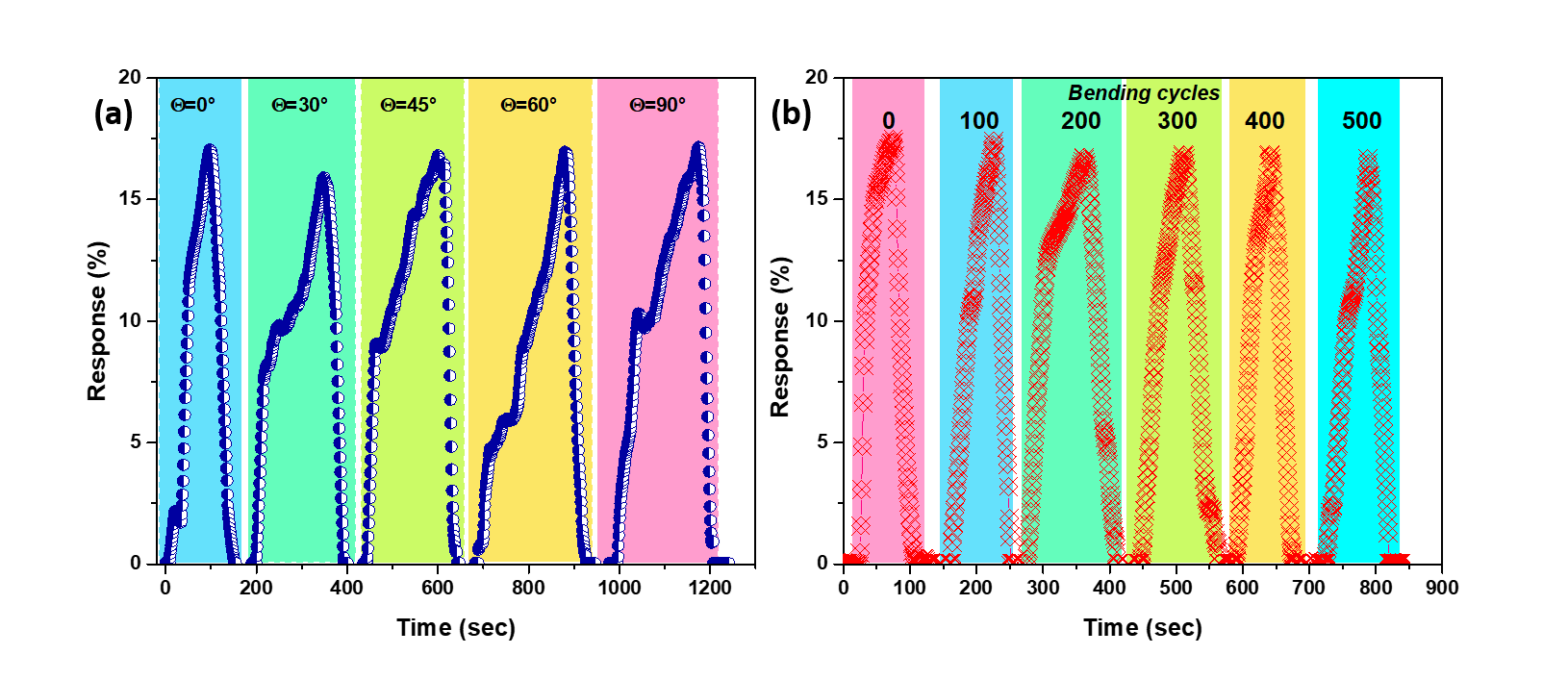


Figure S6 Response curves of 5 wt% NCNO-PPy composite based flexible ammonia sensor at 100 ppm ammonia under (a) different bending angles and (b) upto 500 bending cycles.


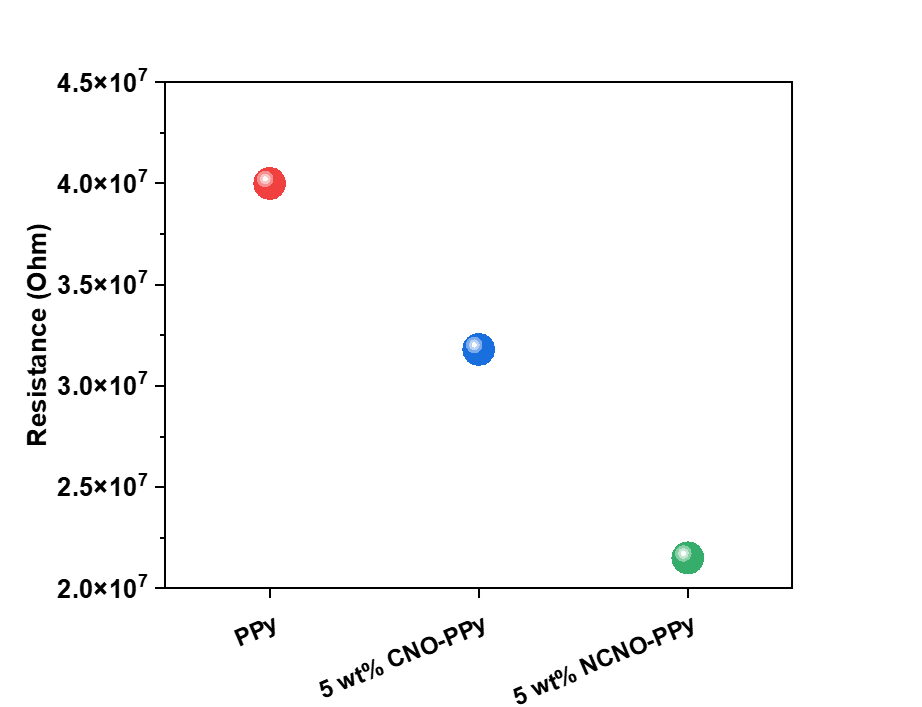


Figure S7 Baseline resistance of pure PPy, 5 wt% CNO-PPy and 5 wt% NCNO-PPy composites.

**Computational Study:**

The computations in this research were performed using the DFT method. The Materials Studio platform DMol3 package was used to carry out all DFT computations. The formation and adsorption energy calculations were performed by applying the Generalized Gradient Approximation through the Perdew–Burke–Ernzerhof method (GGA-PBE), which adopts double numerical basis sets polarization functions (DNP). The electronic properties including HOMO, LUMO and band gap of PPy, CNO, NCNO and PPy-CNO/NCNO composite and their complexes are calculated after the geometrical optimization. The adsorption energy (E_ads_) was defined as follows:

E_ads_ = E_complex_ – (E_material_ + E_ammonia_)……………………..S1

where E_material_, E_ammonia_ and E_complex_ are the total system energy of material, the energy of the gas and energy of total material to gas adsorption complex system, respectively.

For the polypyrrole structure geometrical optimization, three pyrrole rings were taken and all the study was carried out with the same^1,2^. For the optimization of carbon nano-onions structure, fullerene (C-240) was taken^3,4^. Although the carbon nano-onion is a concentric rings of fullerenes with full of defects but for simpler and less computational time study, only one fullerene structure is taken with no defects and then doped with nitrogen atom. For one CNO, maximum three nitrogen atoms were taken for doping purpose^5^. For the PPy-CNO or PPy-NCNO purpose, both the materials were connected though hydrogen bonding after going through previously literature.


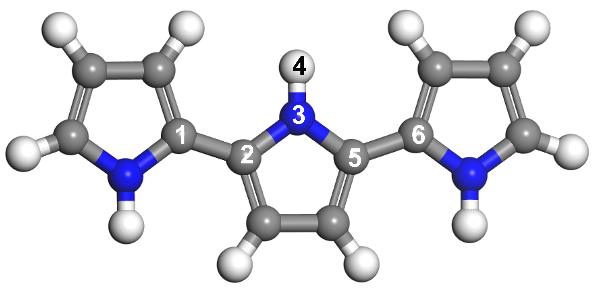


Figure S8 Optimized structure of polypyrrole (3Py).


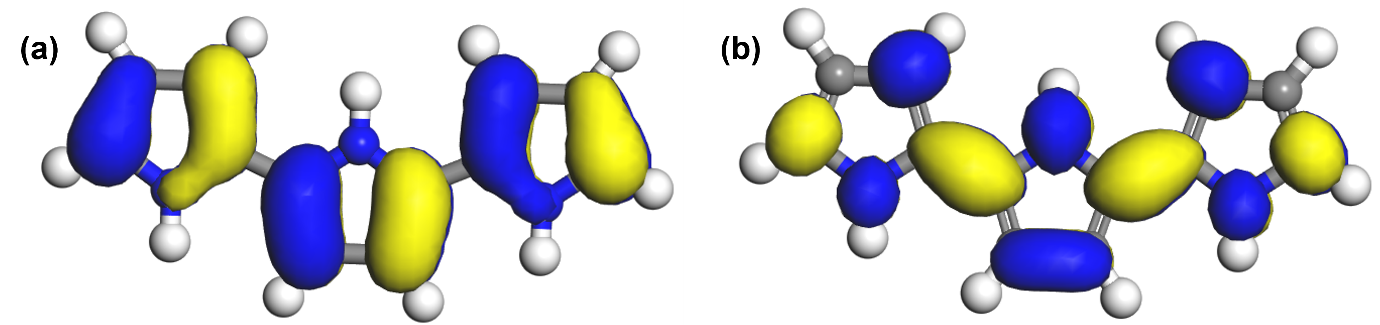


Figure S9 Molecular diagram of 3Py, (a) Highest occupied molecular orbitals (HOMO) and (b) Lowest unoccupied molecular orbitals (LUMO).

At the DFT-DMol3 theory, geometry optimizations of 3Py oligomers were performed. Fig. S8 displays the a reference optimized structure of 3Py. Consequently, frontier molecular orbitals of HOMO and LUMO is presented in Fig. S9 for 3Py. Further, the sensing ability also highly influenced by how an oligomer's molecular orbitals interact with the analyte. Following interaction with the analyte, the sensing material's molecular orbitals—in particular, HOMO and LUMO—become disturbed. Other properties of the sensing substance (polymer in our case), such as band gap, are significantly impacted by this disruption. The geometrical optimized structure of 3Py and ammonia adsorbed 3Py is shown in Fig. S10.The geometrical parameters, which are directly associated with the interaction site such as d (H4-NH3), d (H4-N3) in Å, the angle ∠C2N3C5 and dihedral angle ∠C1C2N3C5 (in deg) is shown in Table S1. As can be seen from the values, there is very little difference in the bond length and angles on interaction with ammonia. This tiny different could be due to the ion dipole interaction in the oligoaniline complexes.


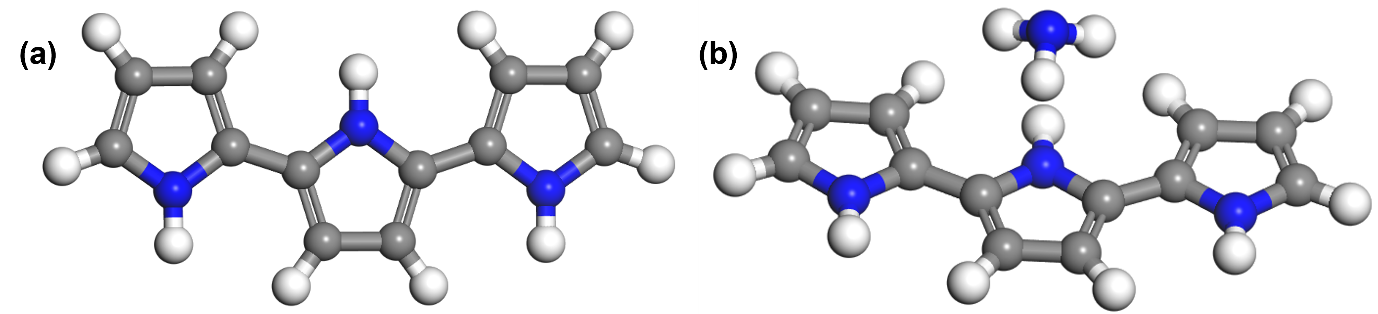


Figure S10 Optimized structure of (a) 3Py and (b) 3Py-NH_3_


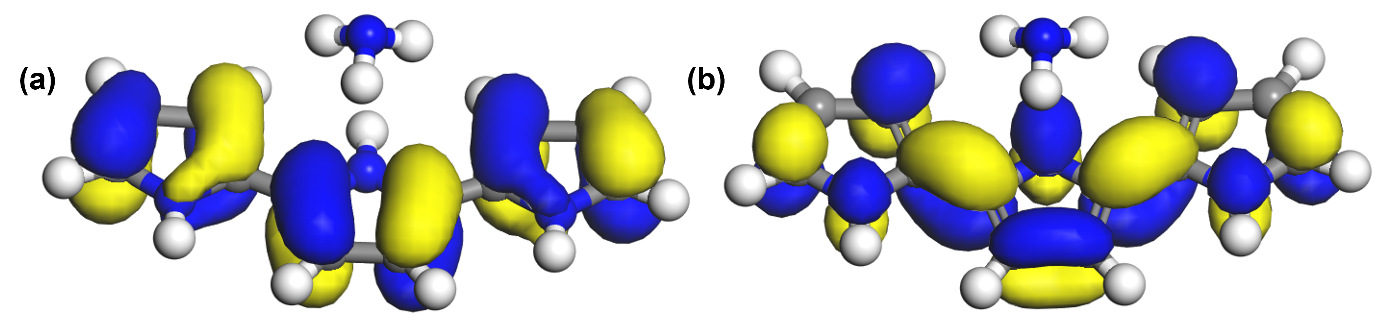


Figure S11 Molecular diagram of 3Py-NH_3_, (a) Highest occupied molecular orbitals (HOMO), and (b) Lowest unoccupied molecular orbitals (LUMO).

Table S3: Geometric parameters of pure 3Py and 3Py ammonia adsorption including d (H_4_-NH_3_), d (H_4_-N_3_) in Å, the angle ∠C_2_N_3_C_5_ and dihedral angle ∠C_1_C_2_N_3_C_5_ (in deg)

| System | d (H_4_-NH_3_) | d (H_4_-N_3_) | ∠ C_2_N_3_C_5_ | ∠ C_1_C_2_N_3_C_5_ |
| --- | --- | --- | --- | --- |
| 3Py |  | 1.109 | 108.617 | 179.849 |
| 3Py-NH_3_ | 1.855 | 1.044 | 110.801 | 178.914 |

Table S4: HOMO, LUMO and Band gap of 3Py and 3Py-NH_3_.

| System | HOMO (eV) | LUMO (eV) | Band gap (eV) |
| --- | --- | --- | --- |
| 3Py | -3.341 | -0.46 | 2.881 |
| 3Py-NH_3_ | -3.031 | -0.161 | 2.87 |


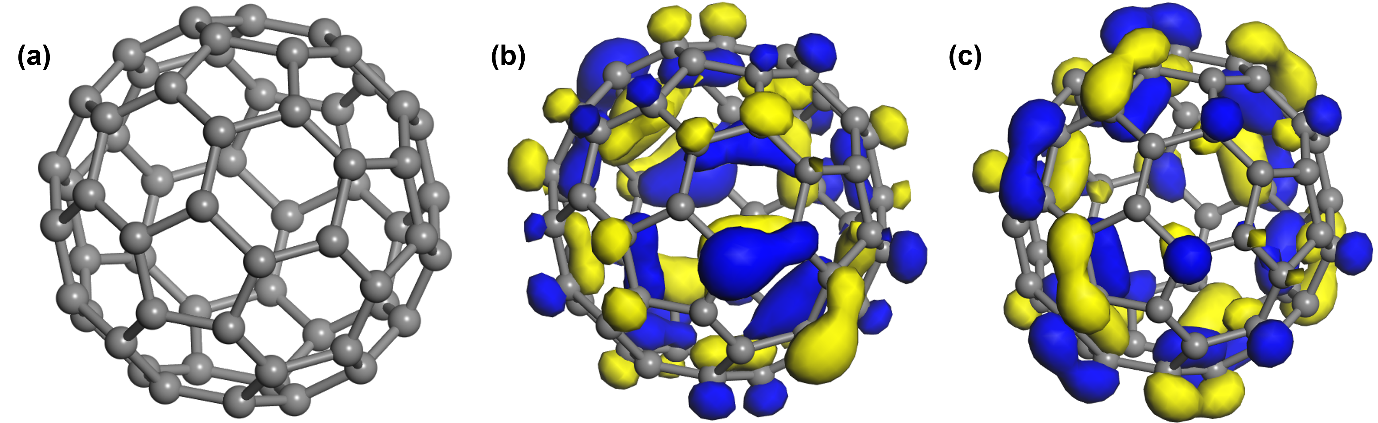


Figure S12 (a) Optimized structure of CNO, Molecular diagram of CNO, (b) HOMO and (c) LUMO.


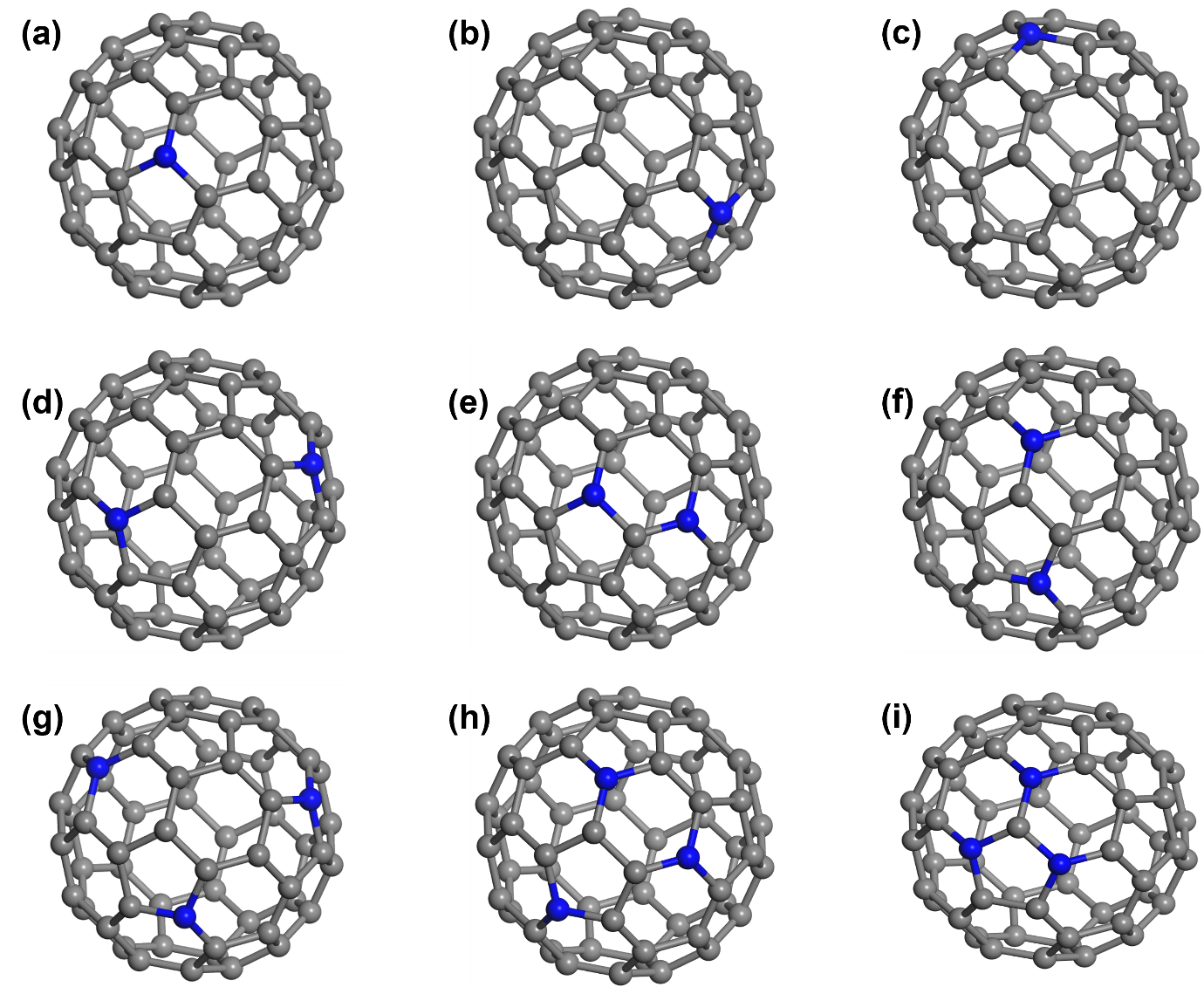


Figure S13 Optimized structure of NCNOs, (a-c) one N atom doped CNO, (d-f) two N atom doped CNO, (g-i) three N atom doped CNO.

Table S5: HOMO, LUMO and Band gap of CNO and NCNOs.

| System | HOMO (eV) | LUMO (eV) | Band gap (eV) |
| --- | --- | --- | --- |
| CNO | -5.149 | -3.516 | 1.633 |
| 1NCNO-a | -5.13 | -3.818 | 1.312 |
| 1NCNO-b | -5.125 | -3.826 | 1.299 |
| 1NCNO-c | -5.13 | -3.818 | 1.312 |
| 2NCNO-d | -3.927 | -3.717 | 0.21 |
| 2NCNO-e | -4.023 | -3.852 | 0.171 |
| 2NCNO-f | -4.018 | -3.754 | 0.264 |
| 3NCNO-g | -4.044 | -3.824 | 0.22 |
| 3NCNO-h | -4.441 | -3.787 | 0.654 |
| 3NCNO-i | -4.057 | -3.569 | 0.488 |


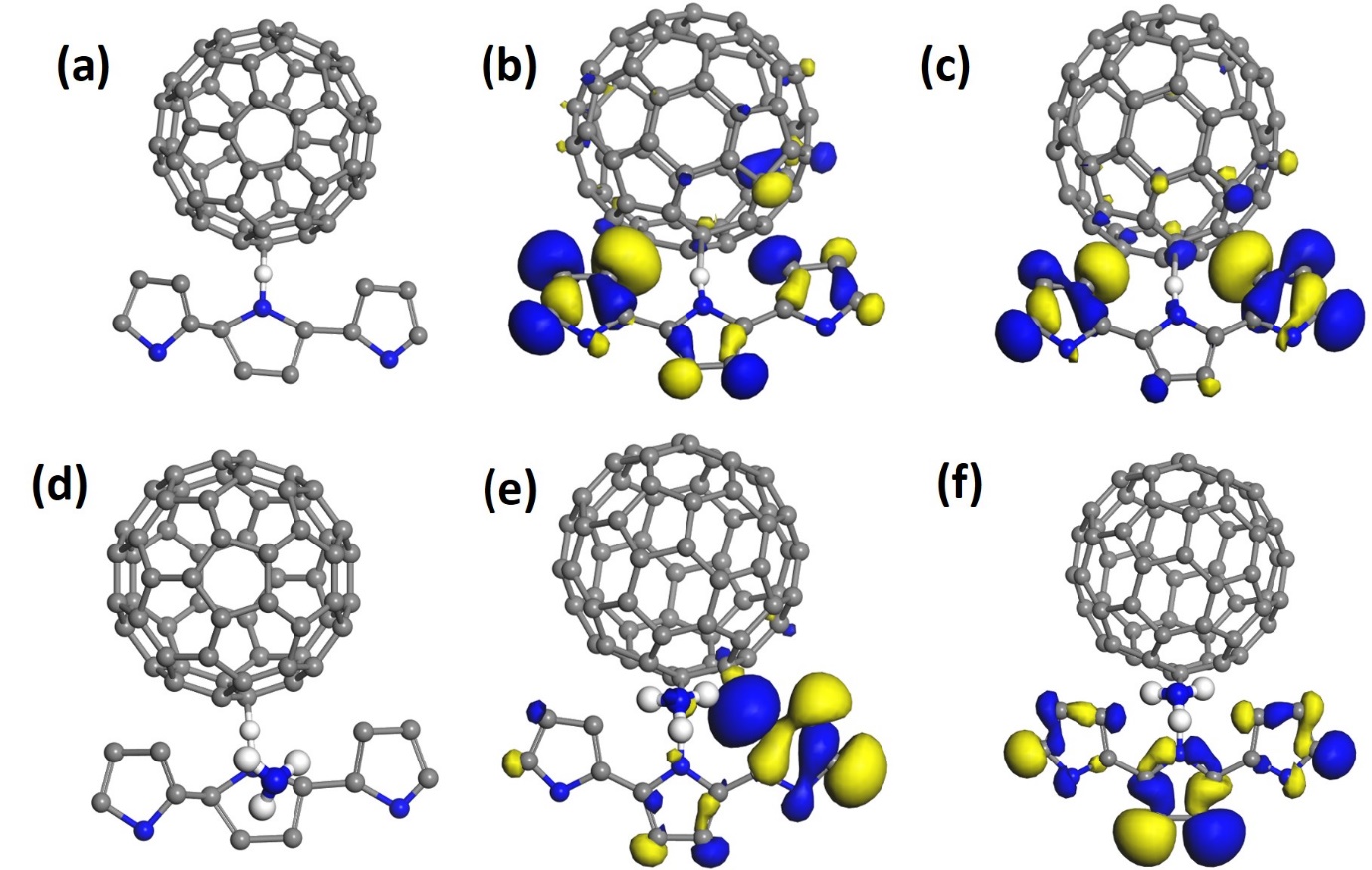


Figure S14 (a-c) Geometrical structure of PPy-CNO composite and HUMO or LUMO orbital, (d-f) Geometry structure of composite with NH_3_ molecular interaction and HUMO or LUMO orbital.


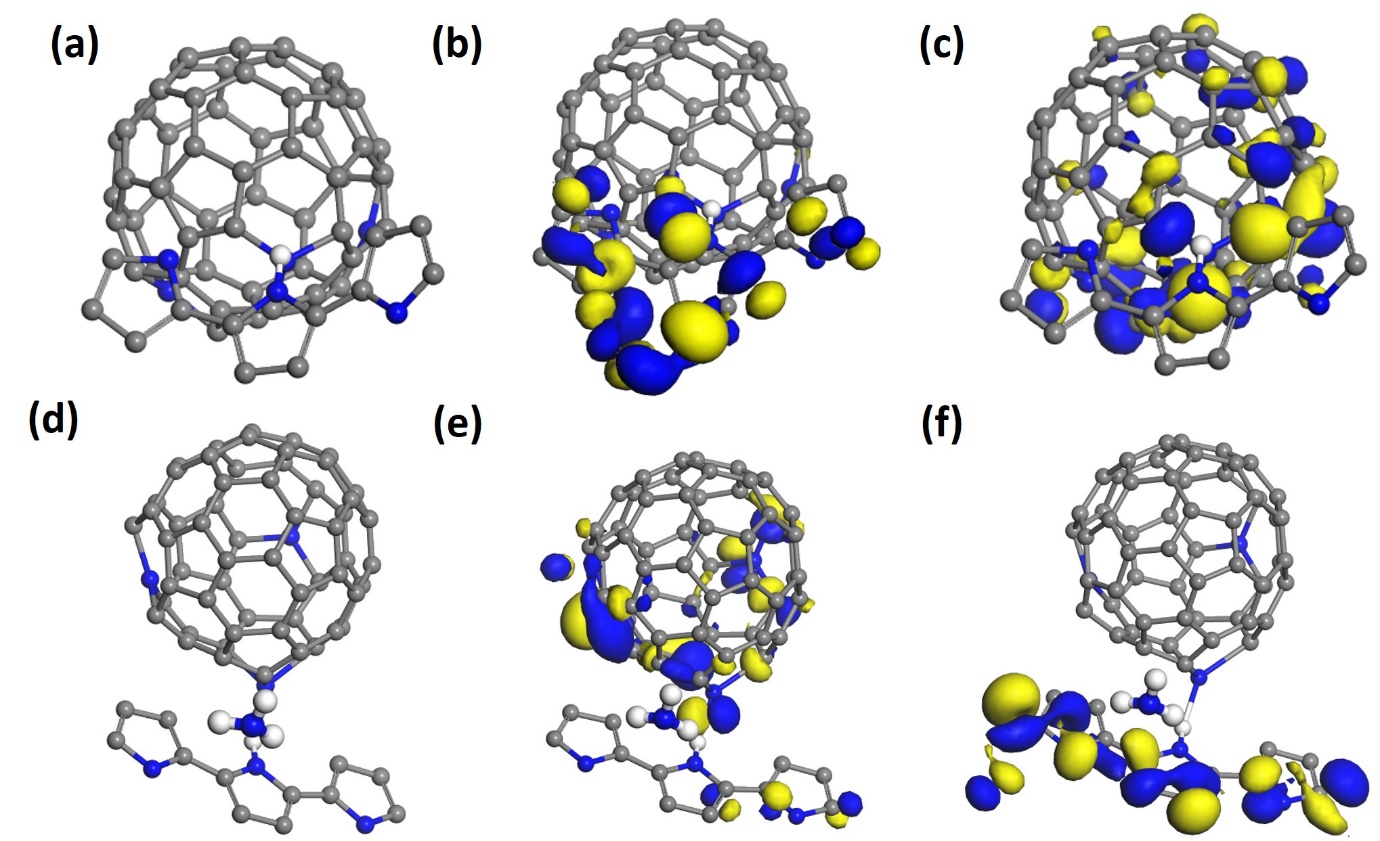


Figure S15 (a-c) Geometrical structure of PPy-3NCNO composite and HUMO or LUMO orbital, (d-f) Geometry structure of composite with NH_3_ molecular interaction and HUMO or LUMO orbital.

**References**

1. Wasim, F., Mahmood, T. & Ayub, K. An accurate cost effective DFT approach to study the sensing behaviour of polypyrrole towards nitrate ions in gas and aqueous phases. *Phys. Chem. Chem. Phys.* **18**, 19236–19247 (2016).

2. Rad, A. S., Nasimi, N., Jafari, M., Shabestari, D. S. & Gerami, E. Ab-initio study of interaction of some atmospheric gases (SO2, NH3, H2O, CO, CH4 and CO2) with polypyrrole (3PPy) gas sensor: DFT calculations. *Sensors Actuators, B Chem.* **220**, 641–651 (2015).

3. Goclon, J. Manipulation of structural and electronic properties of B-doped carbon nano–onions based on DFT modelling. *Appl. Surf. Sci.* **532**, 147267 (2020).

4. Zhang, Z. *et al.* Investigation of ammonia-sensing mechanism on polypyrrole gas sensor based on experimental and theoretical evidence. *Sensors Mater.* **33**, 1443–1454 (2021).

5. Goclon, J., Bankiewicz, B., Kolek, P. & Winkler, K. Role of nitrogen doping in stoichiometric and defective carbon nano-onions: Structural diversity from DFT calculations. *Carbon N. Y.* **176**, 198–208 (2021).

6. Ullah, H. *et al.* Theoretical insight of polypyrrole ammonia gas sensor. *Synth. Met.* **172**, 14–20 (2013).
